# Improving the durability of cobaltite cathode of solid oxide fuel cells – a review

**DOI:** 10.1039/d3ra02571c

**Published:** 2023-08-22

**Authors:** Ali Muqaddas Mehdi, Amjad Hussain, Rak Hyun Song, Tak-Hyoung Lim, Wajahat Waheed Kazmi, Hafiz Ahmad Ishfaq, Muhammad Zubair Khan, SanaUllah Qamar, Muhammad Wasi Syed, Muhammad Taqi Mehran

**Affiliations:** a Hydrogen Energy Research Division, Korea Institute of Energy Research (KIER) 152 Gajeong-ro, Yuseong-gu Daejeon 34129 Republic of Korea; b Department of Advanced Energy and System Engineering, Korea University of Science and Technology (UST) 217 Gajeong-ro, Yuseong-gu Daejeon 34113 Republic of Korea; c Korea Institute of Energy Research, University of Science and Technology 217 Gajeong-ro Yuseong-gu Daejeon 34113 South Korea; d Department of Materials Science and Engineering, Pak-Austria Fachhochschule: Institute of Applied Sciences and Technology Mang Haripur 22621 KPK Pakistan; e School of Chemical and Materials Engineering (SCME), National University of Sciences and Technology (NUST) H-12 Islamabad 44000 Pakistan taqimehran@scme.nust.edu.pk

## Abstract

Solid oxide fuel cells (SOFCs) are highly efficient, low-emission, and fuel-flexible energy conversion devices. However, their commercialization has lagged due to the lack of long-term durability. Among several performance degradation mechanisms, cathode degradation and elemental inter-diffusion of the electrolyte and cathode has been identified as the predominant factors. In the most common SOFC systems, a cobalt-based perovskite material is used, for example LSC or LSCF. These cobalt-based materials offer mixed conductivity and higher concentration of oxygen vacancies as compared to LSM at lower operating temperature leading to favorable reduction kinetics. However, the presence of cobalt results in higher cost, higher thermal expansion co-efficient (TEC) mismatch and most importantly leads to rapid degradation. Various elements like strontium, cobalt, cerium, chromium, or zirconium accumulate or deposit at the electrode–electrolyte interface, which results in sluggish reaction kinetics of the oxygen reduction reaction (ORR). These elements react to form secondary phases that have lower ionic and electronic conductivity, cover active reaction sites, and eventually lead to cell and system deterioration. Over the past decade, several studies have focused on preventative and protective measures to prolong SOFC lifetime which includes novel fabrication techniques, introduction of new layers, addition of thin films to block the cation transport. Such efforts to prevent the formation of insulating phases and decomposition of the cathode have resulted in a remarkable improvement in long-term stability. In this review paper, current research on leading mechanisms responsible for the degradation of cobaltite cathode of solid oxide fuel cell has been summarized and durability improvement strategies of cobalt-based SOFC cathodes have been discussed.

## Abstract 2

Degradation of cobaltite cathode of solid oxide fuel cell and their durability improvement strategies.

## Graphical abstract

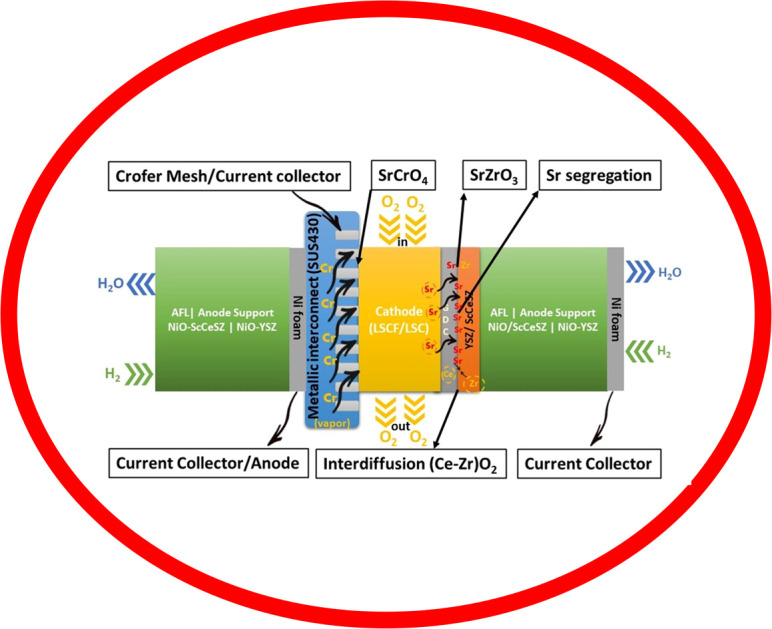

## 1. Introduction

Research on fuel cells is increasingly dominating conventional fossil fuels because of growing environmental concerns. These concerns have spurred efforts to develop renewable energy sources to mitigate climate change. Among the various methods being explored, the development of solid oxide fuel cells (SOFCs) has received substantial traction.^1^ SOFCs offer advantages such as high electrochemical conversion efficiency,^2^ fuel flexibility,^3,4^ and reduced cost.^5^ The application of SOFCs, particularly intermediate temperature SOFCs (IT-SOFCs), have potential in both stationary,^6^ and mobile areas.^7^ For instance, IT-SOFCs can provide advantages such as low noise,^8^ and lightweight properties,^9^ as well as in military applications^10^ when used in unmanned vehicles.^11^ Furthermore, the implementation of IT-SOFCs in stationary applications can advance the concept of microgrids.^12^ To enable the commercialization of SOFCs as a clean energy technology,^13^ extensive research activities have been focused on reducing degradation rates and manufacturing costs.^14^ In our review paper, we specifically address the durability issues associated with SOFCs, aiming to accelerate the commercialization of this versatile technology.^15–19^

**Fig. 1:**
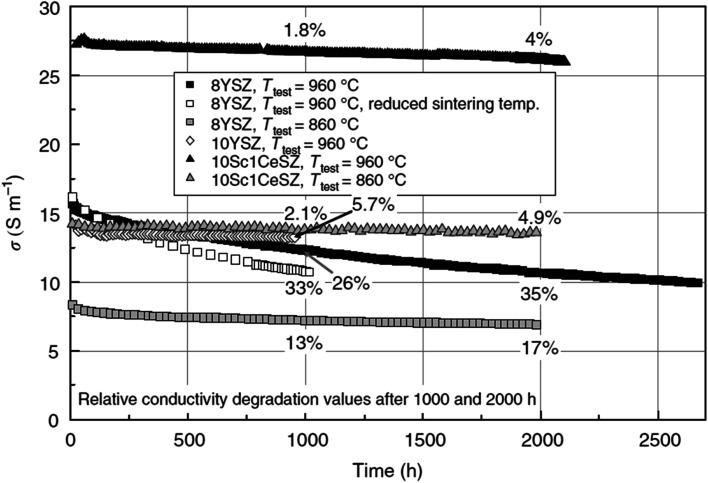
Degradation of conductivity of ceramic materials over a span of 2500 h of continuous testing. (Reproduced with permission from ref. 20 Copyright 2009 Elsevier.)

**Fig. 2:**
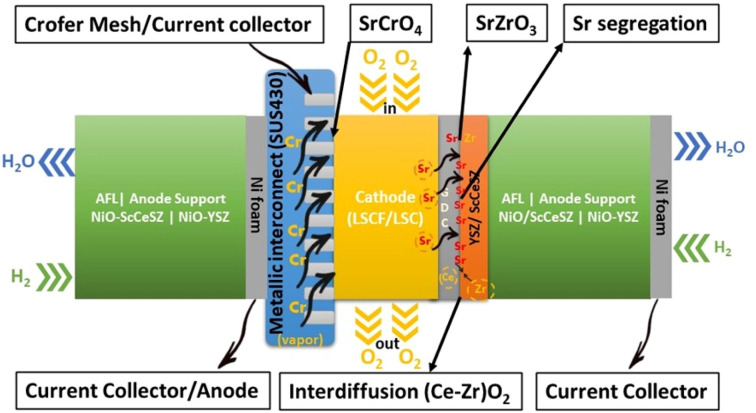
Illustration of degradation issues and their source in a typical SOFC stack.

Fuel cell degradation occurs due to the gradual loss of intrinsic conductivity during operation or the formation of insulating phases. A research study demonstrated that the intrinsic conductivities of various ceramics employed in SOFCs also decline as the operation progresses. Among them, 8 mol% yttria-stabilized zirconia (8YSZ) exhibited the highest conductivity degradation, approximately 35%, over a 2500 hour operation period according to a study^20^ (Fig. 1). Consequently, the selection of suitable materials plays a crucial role in the development of robust fuel cell technology. In addition, in the multi-layer structure of SOFCs, the presence of lanthanum (La) and strontium (Sr) in the cobaltite cathode leads to a reaction with zirconium (Zr) in the electrolyte (either YSZ or ceria-doped scandia-stabilized zirconia (CeScSZ)), resulting in the formation of secondary phases such as lanthanum zirconate (La_2_Zr_2_O_7_)^21,22^ and strontium zirconate (SrZrO_3_)^6^ (Fig. 2). These secondary phases hinder the transport of ions at the electrolyte–cathode interface.^23^ To mitigate this issue, the addition of a gadolinium-doped ceria (GDC) layer between the zirconia-based electrolyte and the cobaltite cathode has proven to be effective in preventing this reaction.^24^ Furthermore, the chemical interdiffusion between the cathode and electrolyte causes significant durability loss during long-term operation.^25^ Thus, the inclusion of a GDC buffer layer between the cathode and electrolyte is essential to address this problem. Unfortunately, many developers of SOFCs still utilize a buffer layer with insufficient film density and a weak interface, allowing substantial elemental diffusion through it.^26^

GDC cannot be densified at such a low sintering temperature due to its refractory nature.^27^ To address this, several researchers have employed sintering aids to achieve full densification of the GDC layer at lower temperatures.^28–31^ However, incorporating sintering aids does not offer a viable solution for the multi-layer fabrication process, as their addition increases reactivity with adjacent components, leading to complex secondary reactions.^31^ Additionally, the rapid shrinkage of the film caused by the sintering aids results in spatially non-uniform densification, residual pores, and weak interface with substrate.^32^ The existence of interfacial pores significantly impacts the durability of SOFCs.^33^ It is incredibly challenging to realize the required structure of electrolyte and GDC layer without interfacial pores.^34^

Furthermore, within a fuel cell stack system, the metallic interconnects also pose a challenge in terms of degradation.^35^ Typically, chromium-based interconnect material called SUS430 is used to connect cells in a stack.^36^ When subjected to high temperatures and exposed to air, these interconnects form a protective oxide coating.^37^ One particular oxide, known as Cr_2_O_3_, evaporates to form chromia vapor, which subsequently reacts with the segregated SrO phase. As a result, SrCrO_4_ is deposited on the cathode interface.^38^ Nevertheless, this phase exhibits poor ionic conductivity, ultimately leading to the deterioration of the cathode and a subsequent decrease in the long-term performance and durability of SOFCs.^39,40^

**Fig. 3:**
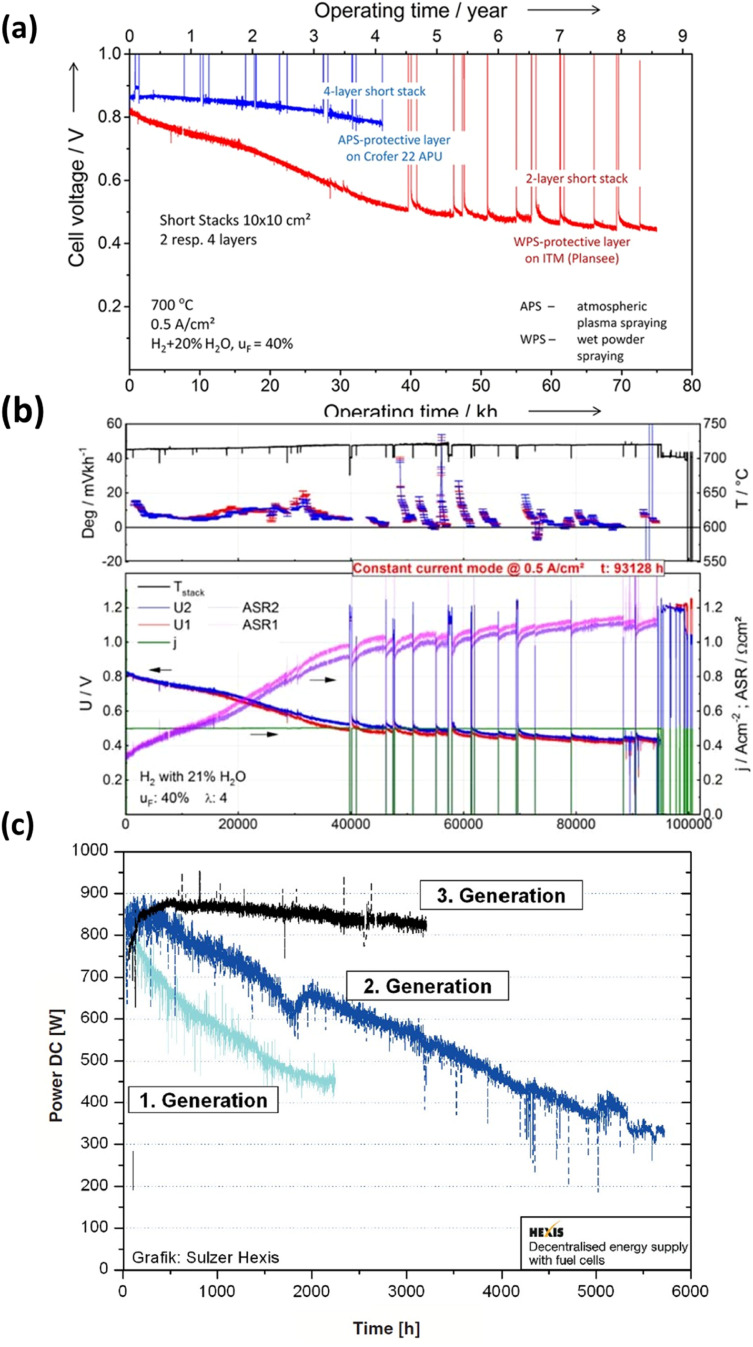
(a) Durability study of short for 70 000 h of operation. (Reproduced with permission from ref. 44 Copyright 2016 Wiley.) (b) Degradation analysis of short stack operated for more than 10 years.^41^ (c) Comparison of degradation curves of first, second and third generation fuel cells.^42^

In recent decades, extensive durability tests have been conducted on SOFC materials and cell structures, aiming to significantly reduce the degradation rate and ensure a prolonged operational lifespan. However, meeting the stringent lifetime criterion remains a challenge: for stationary applications, the fuel cell needs to operate for over 40 000 hours (∼5 years) with a degradation rate of 0.25% kh^−1^.^43^ Blum *et al.*^44^ achieved a remarkable 70 000 hours of continuous operation with an anode-supported SOFC in 2016, reporting a voltage degradation rate of 0.6% per kilohour, as depicted in Fig. 3(a). Three years later, the same research group published results after completing over 100 000 hours of SOFC operation, with 93 000 hours at a constant current density of 0.5 A cm^−2^. Their degradation analysis indicated an overall degradation rate of 0.5% per kilohour for this short stack, showing no discernible pattern or trend in the degradation rate over the 100 000 hour operation period.^41^ Furthermore, Westinghouse^42^ conducted tests on tubular cells for nearly 7 years and observed a degradation rate of 0.4% kh^−1^. Taking it a step further, Frey *et al.* compared fuel cells from 1st, 2nd, and 3rd generation over a 6000 hour period (Fig. 3(c)). Despite these efforts, the benchmark for commercializing SOFC stacks has not yet been achieved. However, it is worth noting that degradation rates varied for single large-area cells. Hussain *et al.*^45–47^ tested a single cell with co-lamination & co-firing method, their findings indicated a degradation rate of 0.2% kh^−1^ for the single cell. 25 A current was applied at 700 °C for 1000 h, and the cell voltage dropped from 0.705 V to 0.703 V (Δ*V* = 0.003 V). Some research groups have also focused on mitigating degradation at the component level. Thaheem *et al.*^48^ employed a protective coating of Mn_1.35_Co_1.35_Cu_0.2_Y_0.1_O_4_ spinel to significantly reduce the area-specific resistance (ASR) degradation of SUS430 interconnects in SOFCs. Their 1000 hour study showed almost no ASR degradation of SOFC interconnects. These encouraging results continue to motivate researchers to further address the degradation issues and transition the technology from single cells to commercial-scale kilowatt (kW) stacks.

Extensive literature reviews have been conducted to understand latest advancements in understanding life-threatening issues of SOFCs. Golkhatmi *et al.*^49^ provided a comprehensive overview of factors that influence the longevity of SOFCs. While they discussed all the components, their approach primarily offered a general perspective on the topic. Sreedhar *et al.*^50^ also examined the degradation issues of SOFCs and offered a detailed examination of the various components and their associated materials as documented in the literature. Similarly, Khan *et al.*^51^ reviewed the degradation of the cathode, ceria interlayer, and electrolyte at the interfaces. They thoroughly discussed and summarized contributing factors, and strategies for improvement. Wang *et al.*^52^ conducted an extensive investigation on sulfur poisoning in SOFC cathodes, analyzing the poisoning mechanism and its relationship with electrochemical performance, while presenting strategies for mitigation. Harrison *et al.*^53^ specifically focused on chromium poisoning in SOFC cathodes and concentrated on manganese-based and cobalt-based cathode materials. This review paper compiles the factors that restrict the lifespan of fuel cell systems incorporating Co-based cathodes, such as La_0.6_Sr_0.4_Co_0.2_Fe_0.8_O_3−*δ*_ (LSCF) or La_0.6_Sr_0.4_CoO_3−*δ*_ (LSC). While cobalt-based cathodes fulfill most requirements for a suitable cathode material, their degradation remains a significant obstacle to commercialization. Consequently, it is crucial to address the degradation specific to Co-based perovskite cathodes. Numerous researchers have conducted experiments with novel fabrication and coating technologies to combat degradation issues in Co-based cathodes, for instance, employing flashlight sintering^54^ or microwave sintering,^55^ using newer/modified materials to achieve better performance and, modifying cathode surface^56^ to bottle-neck the degradation problem at the fundamental level.

**Fig. 4:**
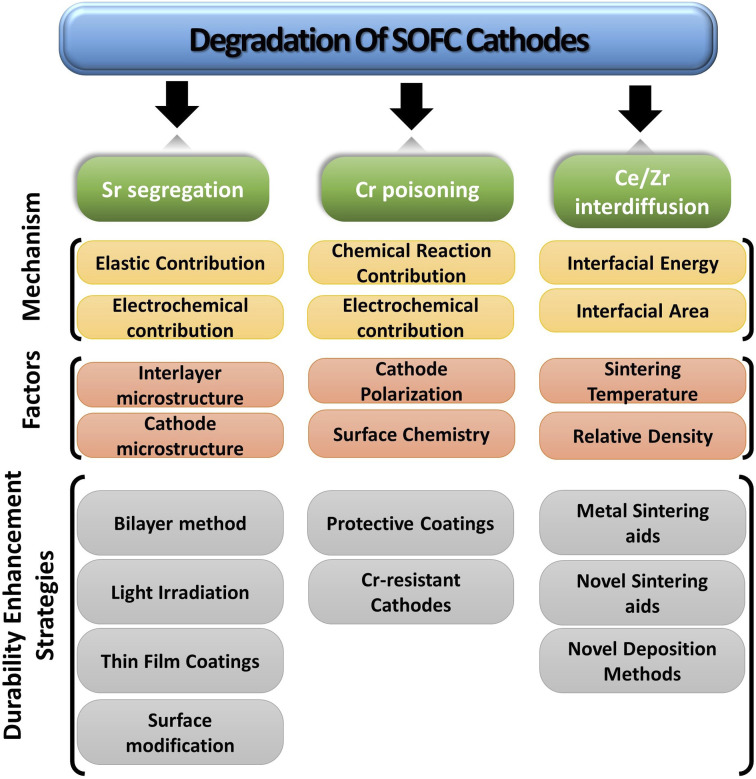
Simplified overview of degradation of SOFC cathodes.

The paper extensively examines the degradation of popular Co-based cathodes (LSC/LSCF) and delves into the underlying phenomena like segregation, deposition and interdiffusion. To better understand the solution strategies, *in situ* and atomic-level studies were incorporated to see how interaction between atoms and ions of these materials lead to Sr segregation, Cr poisoning & ceria–zirconia interdiffusion. Lastly, the durability enhancement methods are presented from literature to gauge progress of commercialization of anode-supported, cobaltite cathode based SOFC (Fig. 4).

## 2. Overview of SOFC

**Fig. 5:**
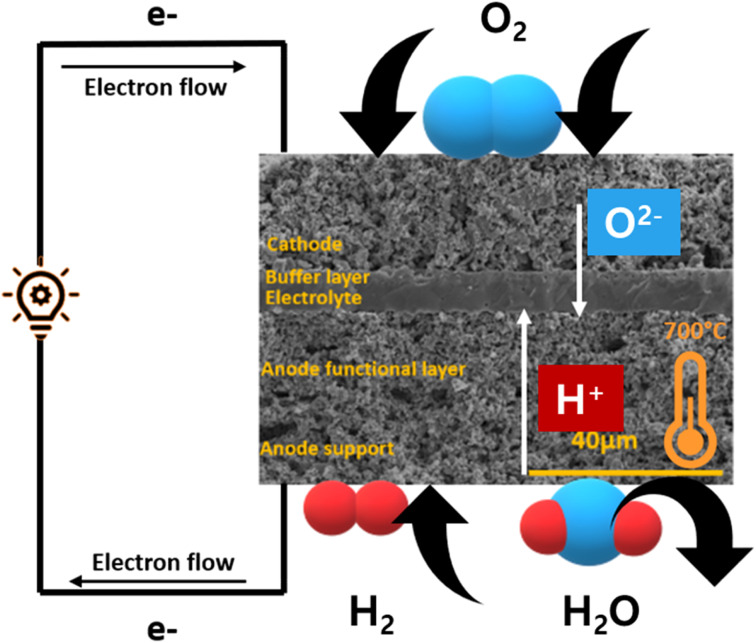
Schematics of a typical O^2−^ conducting SOFC.

SOFC technology consists of high-temperature fuel cell that uses solid electrolyte to convert fuel into electricity^57,58^ (Fig. 5). Hydrogen is supplied at the anode side which moves through the porous, Ni-based anode to react with O^2−^ ions at the electrolyte–cathode interface. A Zr-based electrolyte with high ionic conductivity is typically employed. Air is supplied from the cathode side and water is removed from there as well.

### 2.1. Cobalt-based perovskite cathode material

To achieve a high efficiency in SOFC, the assembly requires a robust cathode material that can offer good reaction kinetics for the oxygen reduction reaction (ORR). This requires high active area and chemical stability in temperature range of 600 to 1000 °C.^59^ The inventors and early developers of SOFC used platinum as an electrode material but it was soon replaced by transition metal oxides as low-cost alternative to a Precious Group Metals (PGM).^59^ In 1966, Button and Archer reported a perovskite-based material as a potential candidate for SOFC cathodes. It soon became one of most widely-studied material for cathodes.^59^

The presence of oxygen vacancies in perovskite material, which remain operational even at elevated temperatures, garnered significant interest of SOFC developers.^59^ These perovskite solids have chemical formula ABO_3_ (ref. 60) where conventionally A-site are doped with rare or alkaline earth metals whereas B-site are occupied by 3d, 4d or 5d transition metals.^61^ Initially, perovskite like LaCoO_3_ and LaMnO_3_ were used. However, the resulting severe degradation with YSZ^62,63^ and ceria-based electrolytes prevented their use for long-term application. Consequently, lower valency dopants like Sr^2+^ or Ca^2+^ were added at A-site to fabricate magnetite and cobaltite cathodes with improved electrical conductivity.^64^ Surprisingly these doped cathodes did not suffer from interfacial reactions with electrolyte as much. Particularly, strontium-doped cobaltite cathodes like LSC demonstrated even superior ion diffusivity and larger surface exchange coefficients.^65^ Additionally, it displayed an even higher tolerance for oxygen vacancy concentration at high operating temperatures.^57^ Therefore, cobaltite cathodes have stayed popular among researchers.

These popular cathodes like strontium doped lanthanum cobalt ferrite (LSCF) possess excellent characteristics: good mixed ionic and electronic conductor (MIEC) and reasonable thermal stability with a TEC that matches with electrolyte/interlayer.^66^ However, these materials suffer from cation migration from bulk to surface when sintered in air at high temperatures.^67^ Such behavior is a major limiting factor in the SOFC long-term stability and durability.^68^

## 3. Degradation of SOFC cobalt-based perovskite cathode material

### 3.1. Sr segregation in LSC/LSCF materials

Since perovskite solid crystals are pushed along a certain direction to make thin films, atoms arrange differently at surface and in the lattice structure. At the surface of the perovskite, cation A has a lower coordination number than in bulk. This leads to different atomic arrangements and thus different levels of free energies in the perovskite material. Moreover, two different kinds of cations with their respective ionic properties exist within the crystal which forces the atoms in bulk to move along a driving force. Segregation essentially relates to the migration of cations from the bulk of cathode to the surface resulting in the enrichment of a particular cation. Therefore, cation A and B segregate out to the surface to form secondary phases. These chemically unstable oxide phases are detrimental to the functionality of SOFC at higher temperatures (>650 °C). Elemental migration degrades an otherwise excellent cathode material over time as the secondary phases have very poor ionic conductivities.

The adverse effects of elemental migration in perovskite-based cathode have been widely reported. In various studies, SrTi_1−*x*_Fe_*x*_O_3_ (STF) surfaces were used as model perovskite system to develop an understanding of surface chemistry relation to reaction kinetics and long-term fuel cell durability.

Jung *et al.*^68^ observed that an excess of Sr on the surface of STF thin film electrodes formed SrO island precipitation, which remained up to two atomic layers. This precipitation was found to impede the oxygen reduction reaction (ORR) kinetics. Mutoro *et al.*^69^ investigated the influence of Sr accumulation on the perovskite surface and its impact on cathode activation or passivation. This poses a challenge in cathode degradation since the increasing Sr content inevitably leads to performance deterioration, without any means of controlling Sr accumulation during operation.

Chen *et al.*^70^ conducted a study demonstrating the detrimental effect of Sr segregation on ORR kinetics in STF thin films. The excessive presence of Sr on the surface hindered the concentration of oxygen vacancies and impeded electron transfer, thereby undermining the reaction kinetics. Additionally, several studies have confirmed the impact of Sr segregation on ORR activity, not only in STF but also in other systems. Cai *et al.*^71^ associated low ORR activity with the surface chemistry of LSC electrodes resulting from high sintering temperatures. LSC electrodes sintered at 650 °C exhibited poor activity due to the presence of Sr(OH)_*x*_ and SrO. Pan *et al.*^72^ validated this correlation by testing two samples under open-circuit voltage (OCV), one with the secondary phases present and the other with the secondary phases removed through chemical etching. In both model systems, such as STF, and thin film cathodes like LSC, Sr segregation leads to a decline in fuel cell performance due to its impact on ORR.

To curtail the threat of Sr-segregation, the phenomenon requires an in-depth morphological examination of the dynamic cathode interface. It forms a thin layer (up to 10 nm as reported by Jung and Tuller^68^) which can be in the form of SrO or other reconstructed phases. In theory, Sr segregates as oxide SrO_*x*_, Sr(OH)_2_·H_2_O or SrCO_3_. Yu *et al.*^73^ reported that secondary phases on LSCF electrode exists as SrO with a capping of SrCO_3_. Subsequently, we can divide the secondary phases into three species: SrO, a reconstructed Sr-excess phase and SrO excess in an ABO_3_ perovskite phase.^74^ There are two major driving factors for Sr-segregation: elastic and electrostatic interactions that may be pushing A-site or B-site cations to move towards the surface.

#### 3.1.1. Role of elastic energy in Sr-segregation

One of the key driving factors behind Sr segregation is the cation size mismatch which induces lattice strain in the perovskite crystal structure. Lee *et al.*^75^ performed an evaluation on the cation size mismatch and associated elastic energy in perovskite structure. To carry out a simplification of elastic interactions, the equation of misfitting solute by Friedel was applied on the perovskite system to have an estimate of the elastic energy.^76^

The equation, though has its own limitations, can provide a good estimate of the elastic energy of dopant, and strengthens our discussion on the dopant size. It was concluded that as the cation size mismatch increases, chemical degradation increases when perovskites were sintered at high temperature in air.^75^

The factor of dopant size along with other factors like fractional free volume of perovskite system,^77^ A-site stoichiometric deficiency^78,79^ reinforces the idea of rearrangement of atoms in perovskite structure. Under high temperature, A-site cation rearrange in a way that accumulation occurs on the surface.^74^

However, elastic energy interaction and relaxation of lattice strain is not the pre-dominant factor in driving cation towards the surface.^74^ The electrostatic interaction between dopant, oxygen vacancies and surface charge play a significant role in the segregation phenomena.

#### 3.1.2. Role of electrostatic energy in Sr-segregation

To understand electrostatic contribution, we first need to understand the ways cathode surface becomes charged. It can happen due to several reasons in an electrochemical system. Firstly, presence of adsorbed gas-phase can induce a charge. Secondly, the loss of symmetry due to the anisotropic nature of cathode material *i.e.*, perovskite causes a redistribution of ions leading a build-up of charge on the surface.^74^

These cathodes develop oxygen vacancies in the bulk and on surface.^80^ These vacancies are higher in concentration at the surface^81^ as coordination number of cations is low. Such a surface carrying a charge leads to an electrostatic driving force for elements to migrate towards the surface, accumulate and form secondary phases. Lee *et al.*^75^ also investigated the electrostatic contribution of segregation along with the elastic one. Simple Coulomb's law generates an electrostatic force for negatively charged dopant to move towards the positively charged surface. Dopant sometimes carries negative charge relative to the lattice when a higher valency A-site cation are substituted, for example when Sr^2+^ is doped in La-based perovskites. Aside from these interactions, morphology and microstructure can also influence the rate of accumulation of these secondary phases.

#### 3.1.3. Effect of interlayer microstructure

As established in the earlier section, GDC interlayer is typically used to curb secondary reactions. The parameters related to GDC like thickness and pore size can significantly impact the segregation phenomena. In our literature survey, a common theme was the thickness of the GDC interlayer. Various researchers have coated thin and thinner layers of GDC and observed the trends.

Constantin *et al.*^82^ investigated the effect of thickness of GDC on cell degradation. As the thickness of GDC decreases, resistance polarization and area-specific resistance decreases, improving LSCF/YSZ half-cell performance. Although, thin GDC layer deposition requires sophisticated techniques, even then thin GDC layer cannot promise improved performance. Khan *et al.*^83^ undertook a similar study and reported the layer of thickness 2.4 µm showed the most promising behavior. Besides the thickness of GDC, fabrication techniques play a fundamental role in addressing segregation issues. Uhlenbruck *et al.*^84^ for example, saw less segregation of Sr when GDC was applied using physical vapor deposition technique instead of the conventional screen printing. Gong *et al.*^85^ also checked the effect of deposition method and found that ion-assisted deposition was better than physical vapor deposition. The improvement in deposition techniques yield highly dense layers of GDC which hinder the migration process and hence improves electrochemical performance.

#### 3.1.4. Effect of cathode microstructure

The microstructure of cathode greatly impacts the level of cation transport. According to reports, porous cathodes have increased cation diffusion toward the GDC interlayer, which leads to the formation of insulating phases. De Vero *et al.*^86^ found that increased Sr diffusion from the LSCF grains to the GDC interlayer caused severe SrZrO_3_ formation at the LSCF/GDC interface in a porous LSCF cathode. The dense LSCF cathode, on the other hand, only had localized SrZrO_3_ grains. The higher barrier stability of GDC interlayer in a dense cathode was attributed to the air-tight cathode–GDC interlayer interface, which reduced microstructural degradation near the LSCF–GDC–O_2_ TPB. The findings of Izuki *et al.*^87^ also revealed limited diffusion of Fe and Co in dense LSCF–GDC diffusion couples. It should be noted that cathode microstructure controls the effectiveness of the contact at the electrolyte–cathode interface. Therefore, it can be concluded that a tight adherence between GDC and cathode can result in limited segregation and eventual durability of the SOFC.

### 3.2. Chromium poisoning from metallic interconnects

Cathode degradation also results from the interconnects used in stacking. Although research continues on newer material for interconnects, but currently metallic interconnects remains a popular choice^88,89^ as ceramic ones are not easy to fabricate. Metallic interconnects help researchers meet large-scale, stationary application needs like residential fuel cell systems of power rating 1 kW, however, their use results in more degradation issues.

Common choice of metallic interconnect includes alloys containing Fe/Cr^90^ or Ni/Cr^91^ and Cr-based oxide dispersed alloys.^36^ At high operating temperatures, these materials form protective oxide scales to prevent oxidation in wet conditions. Mostly, chromia scale along with alumina, silica scale is generated for oxidation prevention, but alumina and silica show very low electrical conductivity (as compared to chromia scale) leading to slow rate of electron transfer, hence a higher ohmic loss. Therefore, chromium-based alloys are preferred in a fuel stack. However, it may crack and delaminate at high temperature when material stresses grow during operation.^92^

**Fig. 6:**
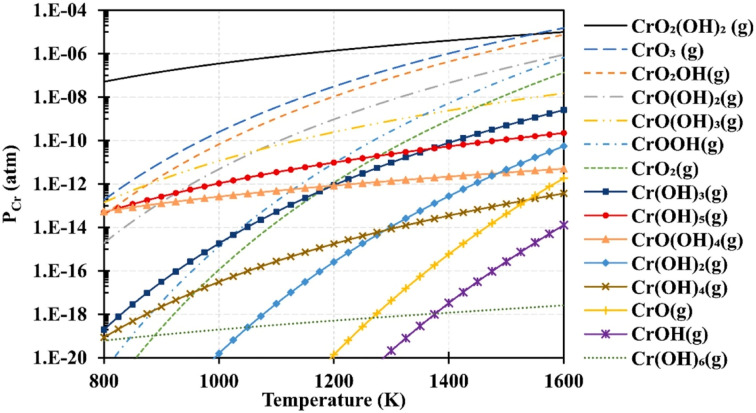
Using Ebbinghaus's thermodynamic data,^93^ partial pressures of several gaseous chromium species over a Cr_2_O_3_(s) source were computed for 1 atm humidified air. (Reproduced with permission from ref. 92 Copyright 2020 Elsevier.)

Based on thermodynamic data presented by Ebbinghaus,^93^ the concentration of chromium-based species that can evaporate from chromia scale at fuel cell operating temperature at 1 atm (humid conditions) was computed (Fig. 6). CrO_2_(OH)_2_ and CrO_3_ vapor dominate the gas-phase mixture that evaporates from the interconnects. These species deposit on cathode as SrCrO_4_ (total conductivity = 1.8 × 10^−4^ S cm^−1^ at 800 °C (ref. 94)) and affects reaction kinetics leading to cathode degradation.^92^

**Fig. 7:**
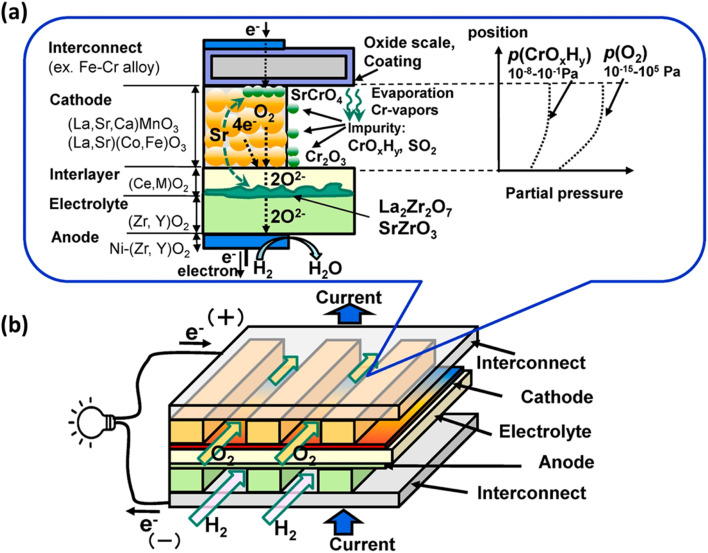
(a) Schematic of the Cr evaporation path and reactions (b) Cr source. (Reproduced with permission from ref. 37 Copyright 2021 Elsevier.)

In particular, gaseous Cr-based compounds cause rapid degradation of cathodes, LSCF.^95,96^Fig. 7 summarizes the source and pathway of Cr evaporation. To develop a fundamental understanding of the degradation mechanism and factor affecting the phenomena, we look at the mechanism prepared by Horita.^37^

These are the reactions that may take place during Cr-poisoning:

• Cr species form from the chromia scale.

• These vapors may deposit on cathode or interlayer and react at the surface.

• Secondary reactions cause element migration of Sr.

• Changes in microstructure of cathode and interlayer occur.

The gas phases present around chromia scale react with it to form Cr-vapors by means of the following possible reactions:

1 Cr_2_O_3(s)_ + 1.5O_2(g)_ → 2CrO_3(g)_

2 Cr_2_O_3(s)_ + 0.5O_2(g)_ + H_2_O_(g)_ → 2CrO(OH)_(g)_

3 Cr_2_O_3(s)_ + 1.5O_2(g)_ + 2H_2_O_(g)_ → 2CrO_2_(OH)_2(g)_

Essentially, the deposition of Cr-based vapor causes elimination of oxygen reaction active sites hindering the ORR at the cathode side. Moreover, these vapors react with segregated Sr species present on the cathode surface to further cause degradation phenomena. Therefore, the mechanism relies on two driving forces: the chemical reaction contribution and electrochemical contribution.

#### 3.2.1. Role of chemical reactivity in Cr poisoning

Thermodynamics suggest that resistance towards Cr-species differ for different perovskites. Yokokawa *et al.*^38^ calculated based on thermodynamic models that LSC/LSCF were least resistant to the deposition of Cr-vapors as compared to the conventional LSM cathodes. This shows that Cr-poisoning behavior differs when A-site or B-site cation type and size changes in a typical ABO_3_.

In the case of LSCF, SrO islands react with Cr-vapors to form Cr–Sr–O nuclei.^37^

4 CrO_2_(OH)_2(g)_ + SrO → (Cr–Sr–O)_nuclei_ + H_2_O

It then grows into SrCrO_4_ and Cr_2_O_3_ in the porous cathode which follows the given reaction:^37^

5 LSCF + *x*CrO_3(g)_ + *x*/2O_2(g)_ → *x*SrCrO_4_ + LSCF

Subsequently, a Co–Fe spinel forms and phase separation occurs.^37^

6 LSCF + *x*CrO_3(g)_ → LSCF + *x*SrCrO_4_ + *y*Co_2_FeO_4_

This reaction scheme ensures that Cr_2_O_3_ vapour reacts to form insulting phases on the cathode surface to impede ORR activity.

#### 3.2.2. Role of electrochemical driving force in Cr-poisoning

One other factor to consider in the promotion of Cr-poisoning is the electrochemical driving force.^97^ Oxygen vacancies on the surface of LSCF electrode are suitable deposition sites for Cr_2_O_3_ vapors. This blocks the reaction sites for the main SOFC operation leading to fast degradation of the cathode.^37^

7 2CrO_3(g)_ + 6e^−^ → Cr_2_O_3(s)_ + 3O^2−^

8 2CrO_2_(OH)_(g)_ + 6e^−^ → Cr_2_O_3(s)_ + 2H_2_O + 3O^2−^

This deposition inhibits the ORR as the reduction of Cr from 6+ to 3+ competes for the same TPB (gas phase/cathode/electrolyte). For LSCF particularly, Cr also deposits at Sr segregated regions where the Cr–Sr–O nuclei formation is favored.

#### 3.2.3. Effect of cathode polarization

Aside from the aforementioned driving forces, other macroscopic factors like cathode polarization also tend to influence Cr-vapor deposition sites. One research group^98^ deliberating flowed Cr-vapors of 10^−3^ P under cathodic polarization of −100 mV and −200 mV with air for 75 h. As we influence deposition sites, higher polarization results in diverse range of product distribution in the bulk and surface of the porous LSCF electrode. The group showed that under higher polarization of cathode, formation of Sr and Cr secondary phase is promoted on the surface while Co–Fe spinel forms as the result of phase separation.

**Fig. 8:**
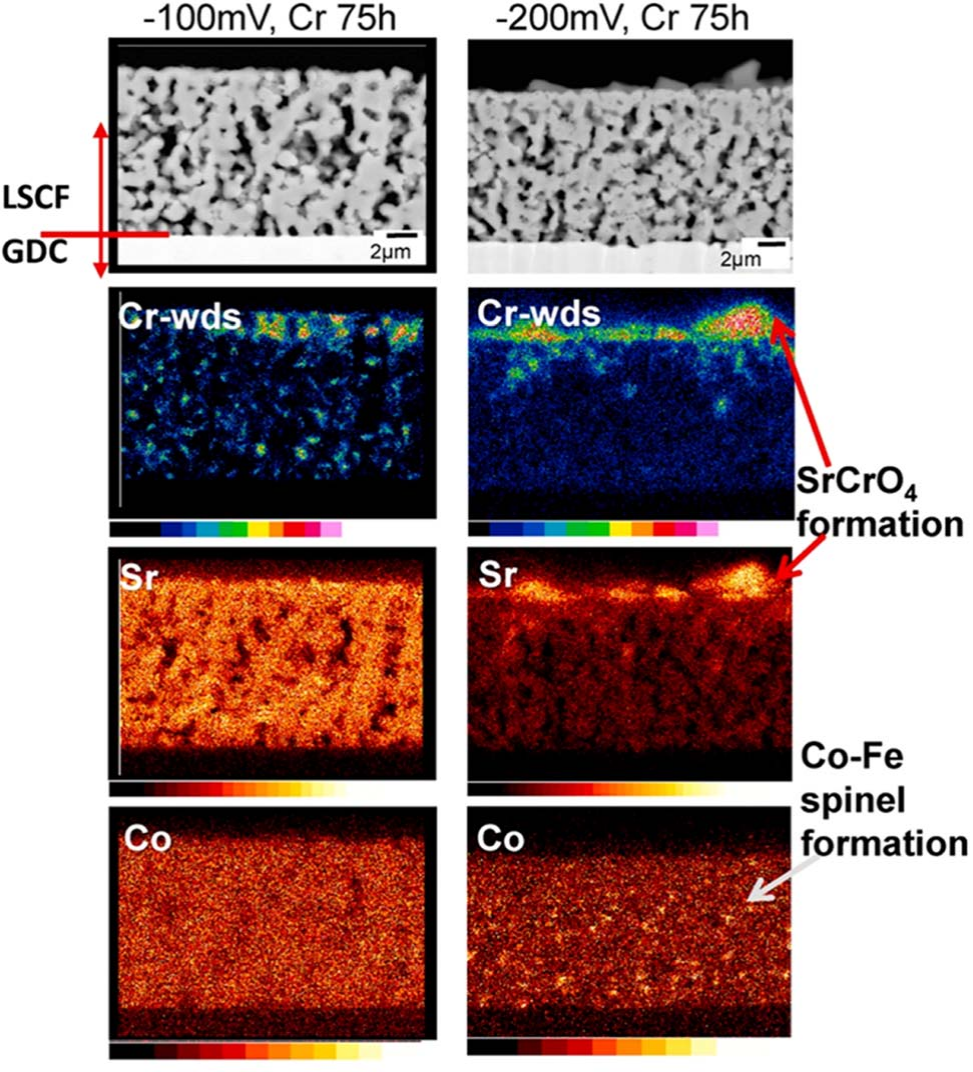
Characterization of cathode at −100 mV potential and −200 mV potential for 75 h to check for Cr content. (Reproduced with permission from ref. 37 Copyright 2021 Elsevier.)

The effect of Cr-based secondary phases on segregation of Sr was observed. Strontium segregated more in a Cr-poisoned cathode due to fast diffusion in the porous LSCF cathode as seen in Fig. 8.

#### 3.2.4. Surface chemistry

**Fig. 9:**
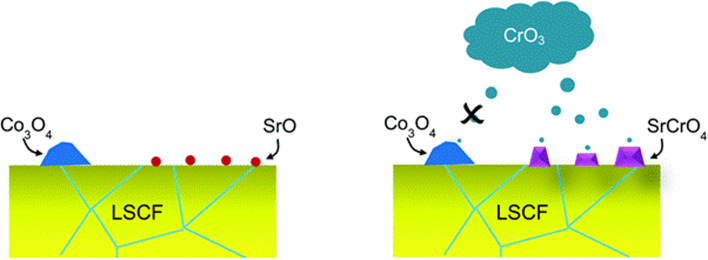
Depiction of the selective nature of Cr-vapor against Co and Sr segregated phases. (Reproduced with permission from ref. 99 Copyright 2014 Royal Society of Chemistry.)

Surface chemistry also play a significant role in poisoning cathode in the presence of chromia scale. Zhao *et al.*^99^ discuss insights regarding the presence of SrO phases and chromium deposition on the LSCF cathodes of SOFC. A study was undertaken on a dense LSCF bar to evaluate the relationship of the presence of SrO islands on the surface and Cr deposition. It was seen (see schematics in Fig. 9) that Cr vapors preferentially deposit on SrO segregated species.

LSCF bar with Cr_2_O_3_ powder was annealed for 96 h in wet and dry conditions. The SEM images showed that in the presence of moisture, LSCF grains change morphologically. On the surface, many small particles form and the surface of LSCF grain is disrupted containing nano-pores or pinholes.

In their findings, both Sr and Co segregated in the form of SrO and Co_3_O_4_ respectively. Further experimentation showed that Cr deposition reaction with Co_3_O_4_ was retarded. Mixing on a molecular level was required for the Cr vapor to react with the cobalt-based secondary phase.

### 3.3. Cation interdiffusion between ceria interlayer and cathode

The phenomena of cation interdiffusion between adjacent electrolyte layers simultaneously affects cathode performance along with segregation & cathode poisoning. It leads to issues like loss of conductivity in the long-term. Normally electrolyte contacts the cathode surface from one side where interfacial reactions may occur. These reactions pose another problem as it leads to adverse secondary components which hinders the conductivity of the electrode/electrolyte material. These interfacial reactions were widely reported in the YSZ/LSCF couple as porous cathode reacts with the dense electrolyte.^100–102^ To block these reactions, the inclusion of ceria-based interlayer^103^ was devised as a strategy. It helped in suppressing cation migration, but newer secondary reactions developed as ceria & zirconia inter-diffused into the adjacent layers.^51^ The most common ceria-based interlayer material used is gadolinium-doped ceria (GDC) film over the YSZ electrolyte.

#### 3.3.1. Densification of GDC

The use of GDC buffer layer imposes two problems. Due to poor sintering characteristics of GDC, it cannot be sintered at a high temperature without the formation of insulating secondary phases. If the buffer layer is sintered at a low temperature, the required relative density is not achieved which promote interdiffusion due to porosity. Here sintering phenomenon is crucial for the required relative density of GDC. To propose a solution, we need to first develop a fundamental understanding of sintering of ceramics.

There are two main driving forces in the sintering process: total interfacial energy and grain growth. As we reduce the total interfacial energy (IE), it results in densification while a reduction in interfacial area (IA) results in grain coarsening or grain growth. IE in the solid-state sintering reduce as solid/vapor are being replaced by grain boundaries (solid/solid interface). Here reduction in IE competes with reduction in IA, thus there exists a trade-off between grain growth and densification.^104^

**Fig. 10:**
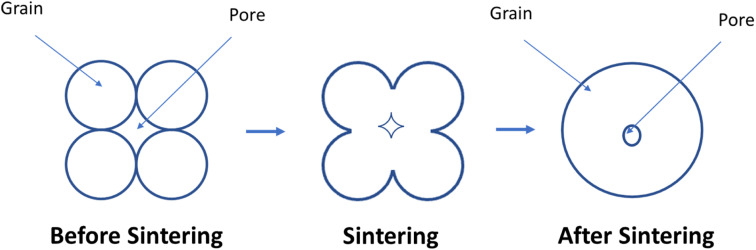
How pore size reduces during sintering process.

The sintering occurs in two main steps. First, grain comes close and form bridge between elemental crystallites which features no shrinkage. Secondly, when residual porosity is eliminated, the ceramic material began to shrink and densify^104^ (Fig. 10).

**Fig. 11:**
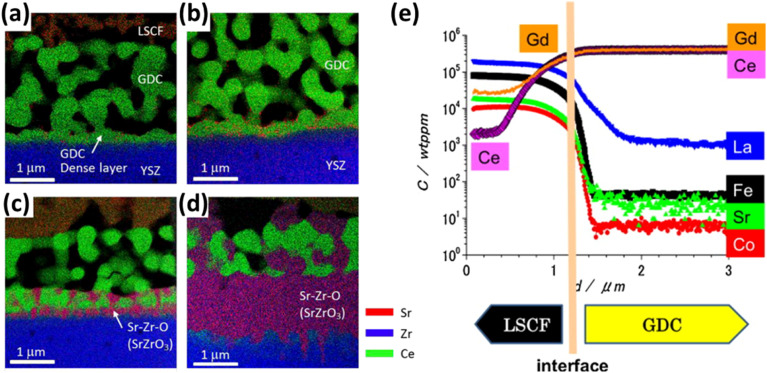
(a)–(d) STEM-EDS of LSCF/GDC/YSZ at 0.5 h, 1 h, 2 h, 16 h to reflect the interdiffusion of Zr, Ce and Sr. (Reproduced with permission from ref. 109 Copyright 2017 Elsevier.) (e) SIMS depth profile of LSCF/GDC diffusion couple to evaluate interdiffusion. (Reproduced with permission from ref. 87 Copyright 2011 Elsevier.)

Although GDC has good ionic conductivity at high temperature, but literature suggests the formation of (Ce–Zr) solid solution at the GDC/YSZ interface while several cations migrate from ceria bulk to the porous LSCF cathode.^105–108^ Izuki *et al.*^87^ experimented with diffusion couples of GDC and LSCF at different temperature to observe interdiffusion. In Fig. 11, SIMS depth profile showed Ce and Gd diffusion into the cathode and La diffusion into GDC.

Similarly, Matsuda *et al.*^109^ characterized the LSCF/GDC/YSZ triplet using Transmission Electron Microscope (TEM) coupled with elemental mapping of Zr, Ce, Sr which revealed the phenomenon of interdiffusion taking place. Wankmüller *et al.*^110^ investigated the YSZ/GDC diffusion couple to see the temperature dependence of the interdiffusion phenomenon. As temperature increased, a thin continuous layer formed whose thickness increased.

**Fig. 12:**
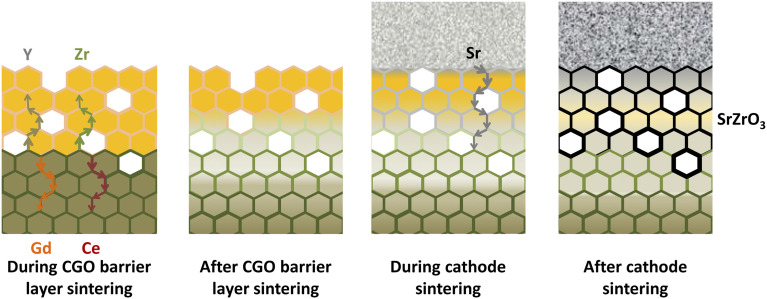
Illustration of the migratory pathways from the GDC interlayer sintering to the cathode and formation of secondary phase. (Reproduced with permission from ref. 51 Copyright 2021 Elsevier.)

This thin secondary layer has poor conductivity (around 1.25 × 10^−1^ S m^−1^) which hinders ion transport at the YSZ interface. Researchers^106^ showed that these cations followed the diffusion path through the free surfaces and grain boundaries as depicted in Fig. 12.

These findings reveal that a ceria-based layer may not be the ultimate solution towards diffusional and migration issues. To work towards the fabrication of a robust fuel cell, it is essential to work with novel techniques to realize the commercialization of SOFCs. Now, we present the current situation of research undertook to mitigate these issues and allow developers to develop kW-scale commercial units.

## 4. Durability improvement strategies of cobalt-based cathodes

Suppressing cation migration, interdiffusion and Cr-poisoning is key to the long-term durability of SOFC. Researchers^56,111,112^ have been trying novel approaches to minimize the effect of segregation and secondary phases to prolong fuel cell operational life.

As already established in Section 2.1, cathode performance is heavily dependent on the suppression of elemental migration. Developing a comprehensive understanding of the factors and driving forces affecting cation migration allows us to theoretically assess the ways to suppress segregation. Several effective methods have been reported in the literature to suppress cation migration into GDC or as secondary phases at the LSC/YSZ interface. These methods explore various fabrication techniques, innovative layering methods and other relevant ideas to hinder elemental migration.

### 4.1. Sr-segregation

#### 4.1.1. Bilayer strategy

**Fig. 13:**
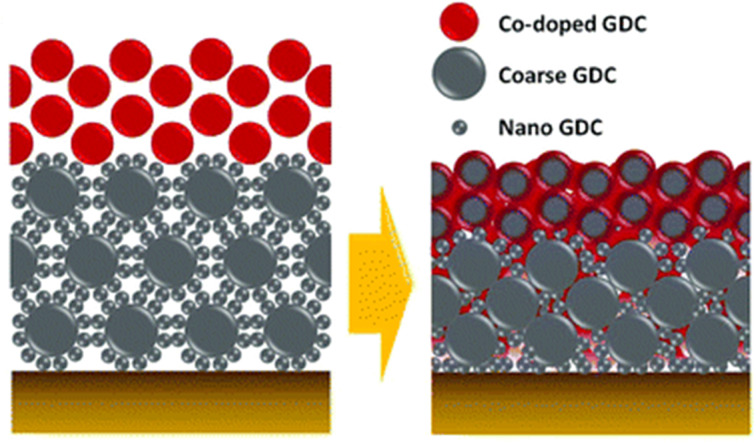
Schematic depiction of the bilayer GDC. (Reproduced with permission from ref. 32 Copyright 2018 Royal Society of Chemistry.)

Many groups have come up with additional layers to counter the diffusion of Sr from bulk LSCF to surface. One such effort was done by Lee *et al.*^32^ who engineering a diffusion-blocking layer by turning GDC into a bilayer (Fig. 13).

**Fig. 14:**
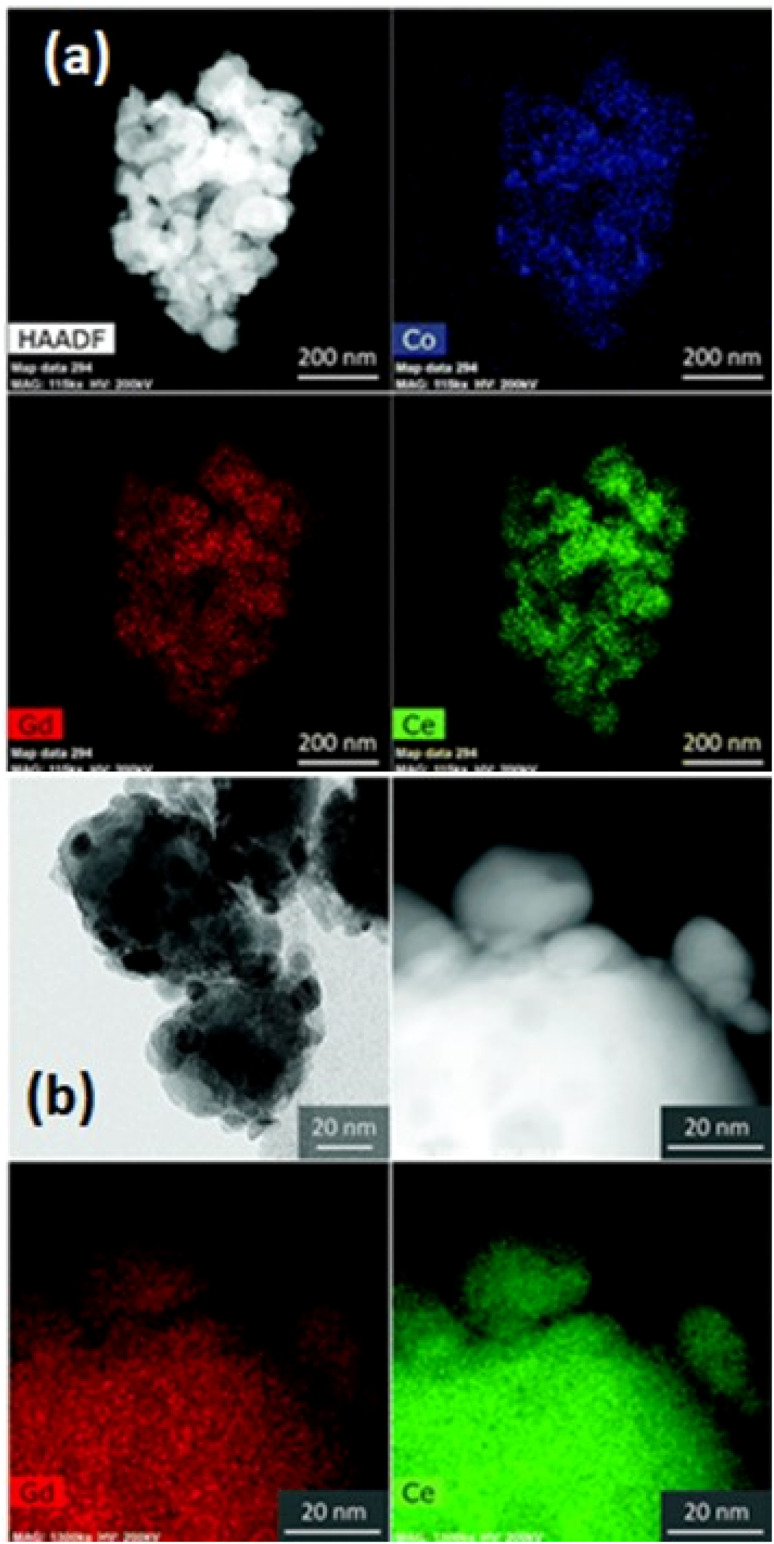
TEM imaging of the morphology and nanoparticle distribution of bilayer from (a) top and (b) bottom. (Reproduced with permission from ref. 32 Copyright 2018 Royal Society of Chemistry.)

This bilayer had different functional top and bottom layer to favor different purposes. The top layer was fabricated using cobalt as a sintering aid and the bottom layer was made from bimodal GDC nanoparticles. Their TEM analysis reveals the morphology and element distribution of the bilayer as shown in Fig. 14.

The top layer contains the sintering aid which facilities the sintering process by generating liquid-like transient phase. Such a phase is highly reactive towards the electrolyte hence a physical barrier in the form of the bottom layer is provided by the bimodal GDC nanoparticles. The transient phase (produced on top layer), which is responsible for densification, migrates to the bottom layer by capillary action to ensure entire bilayer densification. To successfully stop the element migration, the bottom layer needs to be closely packed and highly adhesive. As the film shrinks, the coarse particles in the bottom layer form a continuous matrix phase which imposes local strain suppressing Sr migration. Additionally, the nanoparticles in the bottom layer fill the space between coarse particles to ensure maximum packing density.

**Fig. 15:**
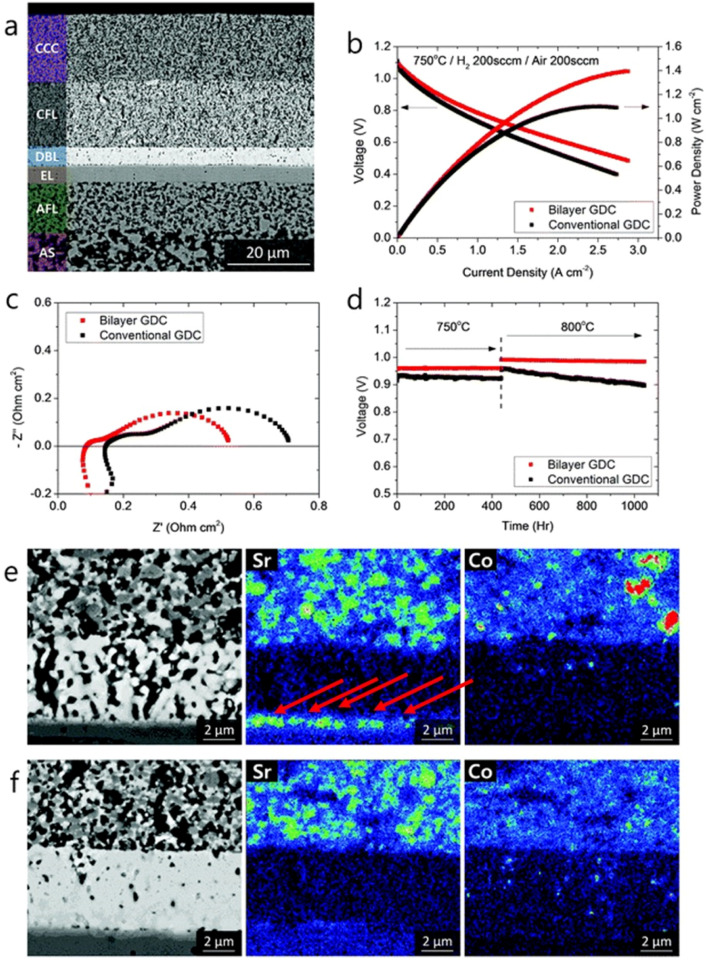
(a) Microstructure of anode-supported cell with bilayer GDC (b) *I*–*V* curves, (c) impedance spectra and (d) long-term evaluation results of the cells with bilayer and conventional GDC. (e) Post-test EDS analysis of conventional cell. (f) Post-test EDS analysis of bilayer cell. (Reproduced with permission from ref. 32 Copyright 2018 Royal Society of Chemistry.)

As for the effect of bilayer approach on electrochemical performance and durability, the results are also effective. The anode supported fuel cell made with the bilayer displayed lower ohmic and polarization resistance. The peak power density observed was 0.78 W cm^−2^ at 650 °C. From Fig. 15(e) and (f), it is evident that Sr segregation is mitigated with the addition of bilayer which is then confirmed from the post-test EDS analysis.

#### 4.1.2. Light irradiation

Most detrimental reactions occur during the sintering process. Park *et al.*^54^ came up with a novel method to limit the use of furnace for sintering and dramatically reduced the sintering time by introducing flashlight irradiation. Typically, sintering process in a process takes more than 20 h to achieve a sintering profile and gradually cools to prevent the cracking of ceramic materials under rapid temperature change.

**Fig. 16:**
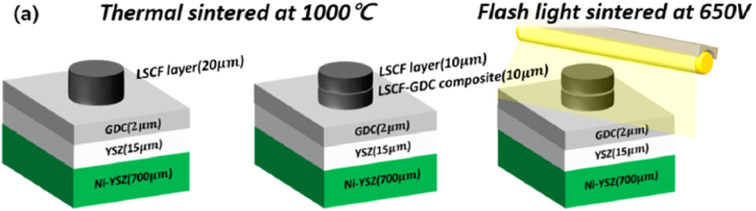
(a) Anode supported LSCF–GDC cathode cell configuration, and flashlight sintering process. (Reproduced with permission from ref. 54 Copyright 2022 Elsevier.)

This light irradiation technique not only minimizes the formation of Sr segregated phase but also dramatically reduce the process fabrication time. The cell configuration is shown in Fig. 16. When sintering temperature of LSCF/GDC composite is increased from 1000 to 1100 °C, particle grow in the direction of pores which reduces porosity while these particles tend to shrink in the direction of layer thickness thus decreasing the thickness of LSCF cathode. Such a phenomenon restricts the performance of cathode during SOFC operation.

On the other hand, when the sample was flashed sintered and later characterized, it showed a reliable morphology that is favorable for fuel cell operation. Sintering at 1050 °C results in a 15 µm cathode/interlayer thickness while flash sintering using 650 V resulted in a much thicker cathode layer of 24.6 µm. Moreover, the interface of cathode and GDC did not blur and remained distinct during the flash sintering process; no secondary phases were generated in the material.

#### 4.1.3. La_0.95_CoO_3−*δ*_ on porous LSCF cathode

**Fig. 17:**
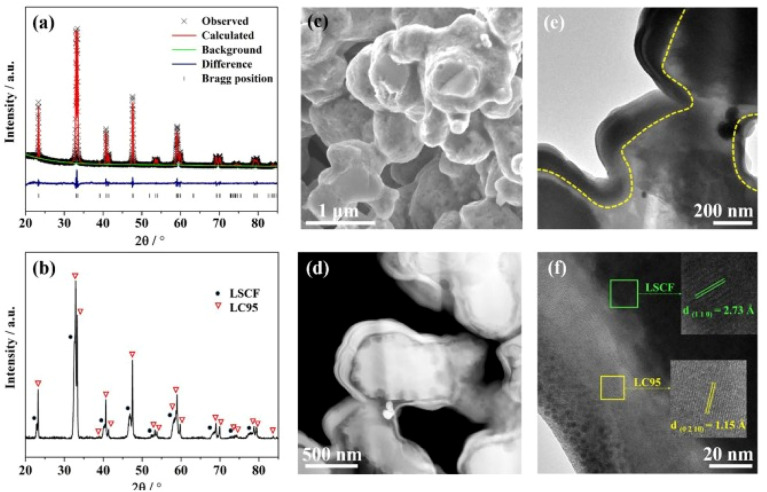
(a) Rietveld refinement spectrum of LC95 powder (b) XRD peaks at 700 °C (c) surface morphology of LC95 coated cathode; (d) HAADF image (e) TEM bright field image of LC95 coated cathode (f) lattice fringe of LC95 coating and LSCF scaffold at high-resolution mode. (Reproduced with permission from ref. 113 Copyright 2022 Elsevier.)

Another method to suppress Sr segregation is to coat an A-site deficient perovskite on the LSCF electrode as carried out by Li *et al.*^113^ A durable, hybrid electrode structure was fabricated with the thin layer coating of La_0.95_CoO_3−*δ*_. The coating was done on the porous cathode using solution infiltration which can form at 600 °C temperature. Li *et al.*^113^ characterized the cathode material to evaluate microstructure and chemical compatibility. Fig. 17 shows the evenly porous structure.

**Fig. 18:**
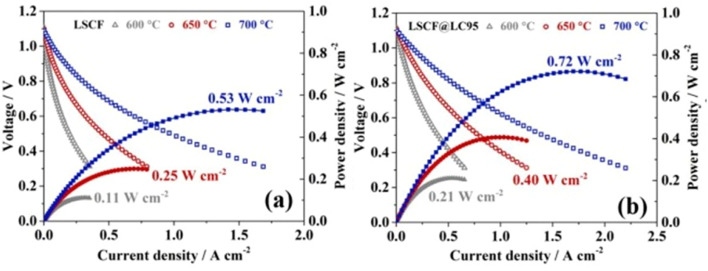
Performance and durability of single cells: *I*–*V*–*P* curves of single cells with (a) LSCF and (b) LSCF@LC95 cathode at intermediate temperature range. (Reproduced with permission from ref. 113 Copyright 2022 Elsevier.)

The *I*–*V*–*P* comparison of LSCF with and without the thin film coating revealed good correlation between electrochemical performance with the presence of La_0.95_CoO_3−*δ*_ as shown in the Fig. 18.

Such a thin film acts as solid solution to accept diffused Sr species to stop the segregation. Moreover, La_0.95_CoO_3−*δ*_ possesses high electronic conductivity thus facilities charge transfer on cathode surface. It further supports the reaction kinetics of the air electrode. Overall, cells with LSCF and La_0.95_CoO_3−*δ*_ display good long-term stability.

#### 4.1.4. PrNi_0.5_Mn_0.5_O_3_ (PNM) thin film with ex-soluted PrO_*x*_ nano particles

**Fig. 19:**
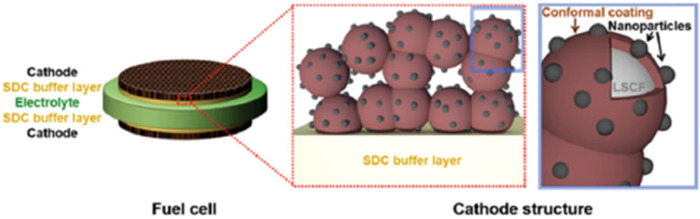
Schematics of an LSCF electrode backbone with nanoparticles. (Reproduced with permission from ref. 114 Copyright 2017 Royal Society of Chemistry.)

Chen *et al.*^114^ investigated a vacancy-rich thin film coat to improve the electrochemical performance of SOFC cathode material and suppress Sr segregation. PNM thin film with exsoluted PrO_*x*_ nanoparticle was used to increase performance whilst maintaining cell durability. This hybrid catalyst was made using one-step infiltration and coated it using PLD as shown in Fig. 19.

**Fig. 20:**
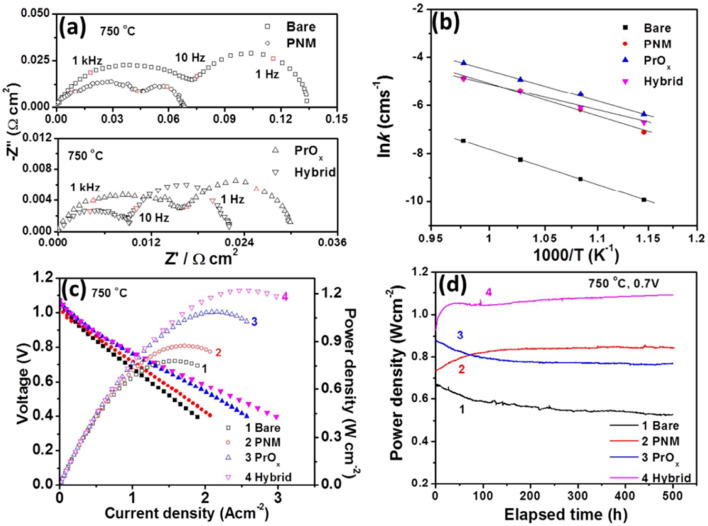
(a) Impedance spectroscopy of bare cathode and PMN cathode (b) conductivity plot (c) *I*–*V* curves (d) peak power density over time. (Reproduced with permission from ref. 114 Copyright 2017 Royal Society of Chemistry.)

Their findings showed the PNM with exsoluted nanoparticles displayed similar characteristics as that of LSCF and hence proved a good candidate for Sr suppression. The hybrid catalyst coated LSCF cathode showed 6 times lesser cathode polarization resistance than that of the regular LSCF cathode while showing excellent peak power density (1.21 W cm^−2^) and durability (0.7 V for 500 h) (Fig. 20).

#### 4.1.5. Less reducible cations (Hf^4+^)

Another interesting approach towards cation migration is to devise methods or techniques that directly hinder the fundamental driving force of Sr segregation. As discussed earlier, the presence of positively charged oxygen vacancies attract the (relative) negatively charged A-site cation towards the surface to form secondary phases like SrO islands.

Tsvetkov *et al.*^56^ came up with a method to reduce the number of oxygen-vacancies to halt the segregation process. It is noteworthy that oxygen vacancies are pivotal for the reaction kinetics and ion transport, however, their work revealed a valuable insight.

**Fig. 21:**
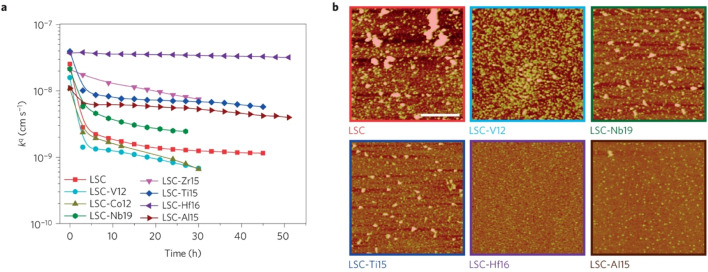
(a) Oxygen surface exchange coefficient, *k*^q^, quantified from electrochemical impedance spectroscopy measurements over time at 530 °C in air, for the LSC and LSC-Me films. (b) Atomic force microscopy images of the LSC, LSC-V12, LSC-Nb19, LSC-Ti15, LSC-Hf. (Reproduced with permission from ref. 56 Copyright 2016 Nature.)

**Fig. 22:**
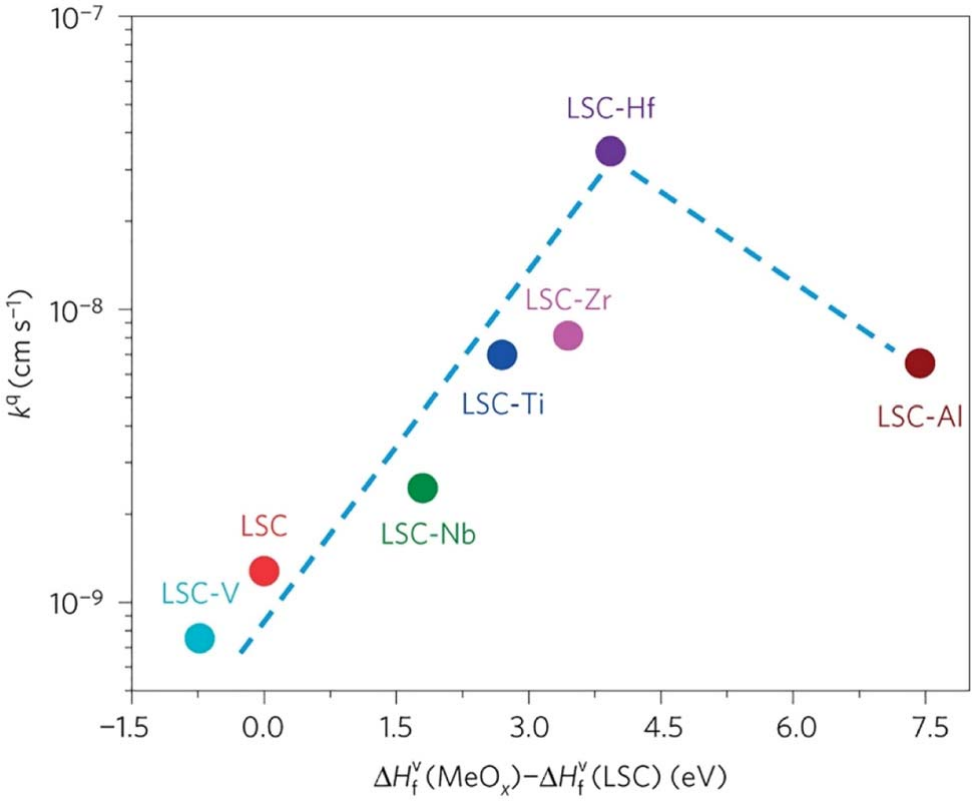
Correlation of oxygen surface exchange kinetics on the reducibility of the LSC surface. (Reproduced with permission from ref. 56 Copyright 2016 Nature.)

The group found out that reducing the concentration of oxygen vacancies is favorable for both segregation and reaction kinetics as clearly shown in *ex situ* Atomic Force Microscopy (AFM) images in Fig. 21. A volcano-like graph trend (Fig. 22) was observed which suggested that an optimum concentration of vacancies can achieve a fine balance between suppression of elemental migration and favorable reaction kinetics.

### 4.2. Chromium evaporation

#### 4.2.1. Cu–Mn spinels

To stop chromium evaporation, many researchers have placed protective coatings over the metallic interconnect. These coatings need to be resistant to strong oxidation environment even at high temperatures. To select a material for such a task, activation energy must be as low as possible, so it remains inert and stable.^115^ Furthermore, it should be a good electronic conductor to keep current collection uninterrupted. These coatings must not allow oxygen to diffuse into their structure which will cause degradation in fuel cell operation.

Cu–Mn, or Co–Mn spinel seem to be promising materials for interconnect coating.^116^ Wei *et al.* reported that the following spinel Co_3_O_4_, CuFe_2_O_4_, Mn_*x*_Co_3−*x*_O_4_ and Cu_*x*_Mn_3−*x*_O_4_ displayed reasonable thermal and electrical properties.^116^

One group of researchers^117^ choose Mn_3_O_4_ and CuMn_2_O_4_ spinel and characterized it using powder X-ray diffraction and scanning electron microscope. A spinel coating over SUS430 interconnect was placed in a muffle furnace at 800 °C for 1000 h; the samples were removed after every 100 h to undergo Energy Dispersive Spectroscopy (EDS) and FESEM. In addition, area specific resistance was calculated to compare it to the uncoated samples. Their findings showed that coating SUS430 with Mn_3_O_4_ and CuMn_2_O_4_ spinel prevented chromium deposition on active sites of the cathode. The microstructure of the coated and tested samples revealed only minor Cr content was found after oxidation for 1000 h and small cracks opened due to outward diffusion of Cr from the interconnect.

**Fig. 23:**
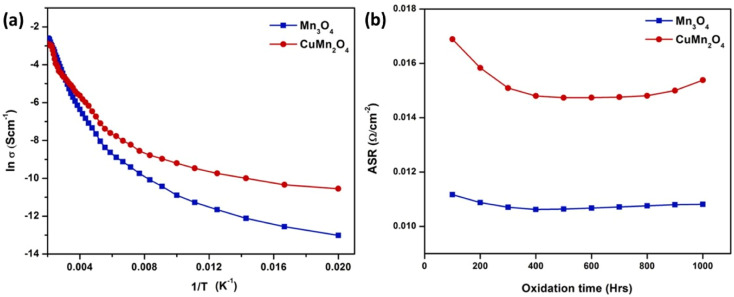
(a) Arrhenius plot shows CuMn_2_O_4_ has higher electrical conductivity (b) ASR measurement of Mn_3_O_4_ and CuMn_2_O_4_ coated SUS430. (Reproduced with permission from ref. 117 Copyright 2022 Elsevier.)

Both spinel materials showed good electronic conductivity over a range of temperatures with CuMn_2_O_4_ showed a slightly improved conductivity behavior. Acharya *et al.*^117^ attributed this result to addition of Cu as it creates voids resulting in better carrier conduction mechanism. Moreover, these materials maintained their ASR over the period of 1000 h with a slight decrease in the case of CuMn_2_O_4_ spinel as depicted in Fig. 23.

#### 4.2.2. Electrophoretic deposition

Various methods have been used to deposit a protective layer for the SUS430 interconnect material.

The simplest one has been discussed previously but there are other novel methods such as sol–gel dip coating,^118^ spray coating,^119^ sputtering,^120^ and electrophoretic deposition.^121^ One group of researchers^122^ explored the electrophoretic deposition (ED) technique to coat a spinel over the stainless-steel substrate, characterized it and tested in air at very high temperatures. To use ED, a 2 mm-thick 460FC stainless steel interconnect with 30 mm to 10 mm substrate dimensions was utilized. Charged powder of Cu_1.35_Mn_1.65_O_4_ spinel in dispersed into iodine-dissolved ethanol to form suspension. The stainless-steel interconnects were then immersed into the suspension and DC voltage (20–60 V) was applied for 30–120 seconds with a gradual increments every 30 seconds.

**Fig. 24:**
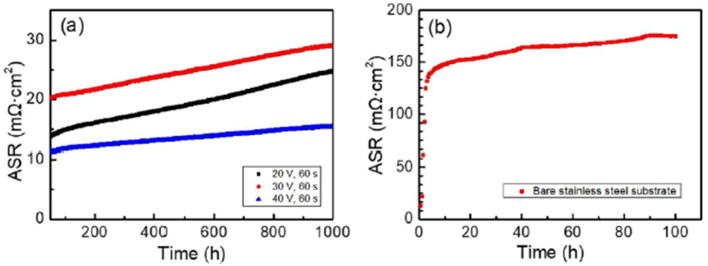
(a) *In situ* monitored ASR values for spinel coated samples at 700 °C for up to 1000 h and (b) ASR of the uncoated 460FC stainless steel substrate, measured under a current load of 10 mA cm^−2^. (Reproduced with permission from ref. 122 Copyright 2022 Elsevier.)

For the testing procedure, the spinel coated substrate was heat treated to enable low-temperature densification by means of reconstruction. In a reducing environment, the spinel was converted to Cu metal and MnO at 800 °C. This was again reconstructed as a spinel using a solid-state reaction at 700 °C in air (cathode side condition). This group reported a dense, uniform spinel coated interconnect which showed very good ASR values (attached in Fig. 24) as compared to bare stainless-steel interconnect.

#### 4.2.3. Electroless silver coating

Another method to prevent Cr poisoning is to use sacrificial coating over cathode material so formation of SrCrO_4_ as the result of Cr poisoning can be hindered. This technique is different from protective coating over the interconnect material as it does not stop Cr evaporation instead prevents cathode degradation from Cr vapors. Wang *et al.*^123^ introduced silver plating over the cathode structure to inhibit the adverse effects of Cr-poisoning. A Sm-doped, ceria-based (Sm_0.2_Ce_0.8_O_1.9_) anode support material with NiO as AFL was fabricated and LSCF plated with Ag was used as the cathode material for cell testing. Silver was plated using electroless technique by means of 1 M silver ammonia solution. This solution was added dropwise on LSCF cathode and then reducing agent was supplied to complete the reaction. It was later calcinated at 150 °C for 1 to remove water content from it.

**Fig. 25:**
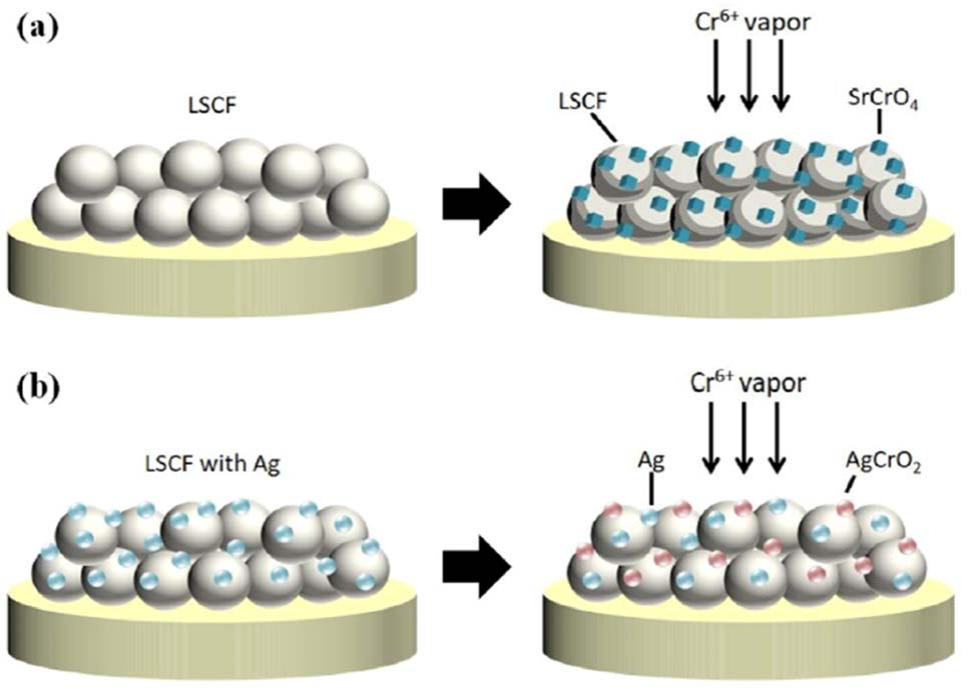
Depiction of (a) Cr-secondary phase formation without cathode modification (b) Cr-resistance with LSCF–Ag cathode. (Reproduced with permission from ref. 123 Copyright 2022 Elsevier.)

**Fig. 26:**
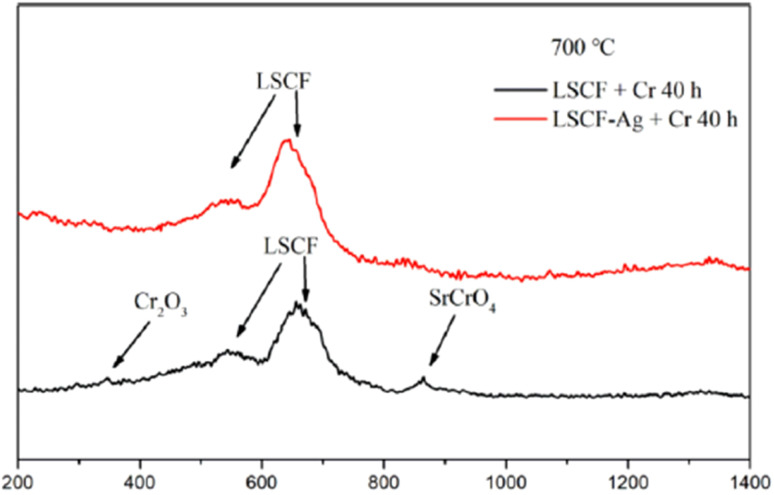
Typical Raman spectra of LSCF and LSCF–Ag cathodes after the Cr treatment at 700 °C for 40 h. (Reproduced with permission from ref. 123 Copyright 2022 Elsevier.)

The choice of silver is also good as it possesses good electrical conductivity and catalytic properties for ORR at the cathode side. Fig. 25 displays the mechanism of Ag plating to mitigate cathode poisoning. This electroless plating offers a reaction site for Cr vapor to deposit and form AgCrO_2_ (see Raman spectra in Fig. 26) which has higher electronic conductivity than SrCrO_4_. The formation of Ag–Cr compound instead of the Sr–Cr alleviates the problem of degradation significantly. Single cells made from Ag-plated LSCF showed 12 percent higher peak power density and proved to be an effective method to curtail Cr poisoning.

### 4.3. Cation interdiffusion

#### 4.3.1. Sintering aid (Li/Cu/Zn)

Sintering aids have been a common way to overcome the densification problem of GDC interlayer. Among the sintering aids, metals are a popular choice.^28^ However, these sintering aids promote other interfacial reactions, so research has been carried out to improve sintering aids or apply other techniques. In one such work by Nicollet *et al.*,^31^ Li, Cu and Zn were added to GDC as a sintering aid. The densification of the interlayer and electrochemical performance of cells improved significantly. Metal nitrates in ethanol (Li/Cu/Zn) were added GDC powder, dried, and prepared as ink to screen-print on YSZ electrolyte.

**Fig. 27:**
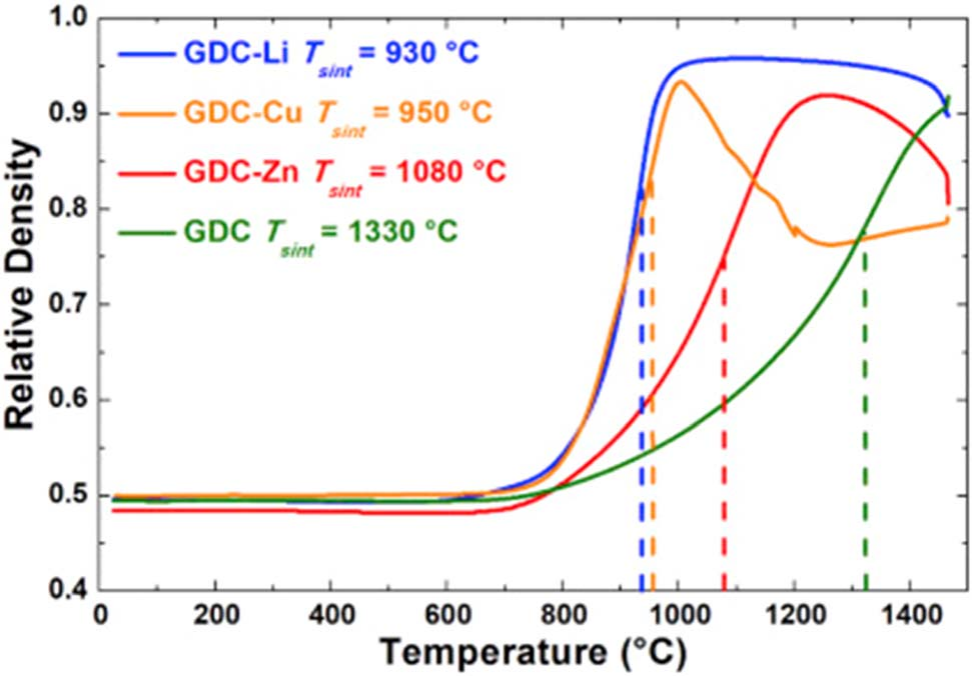
Sintering curves of GDC without additive and with Li, Cu, or Zn. (Reproduced with permission from ref. 31 Copyright 2017 Elsevier.)

The relative density of GDC with and without the sintering aids were compared over a temperature range of 700–1500 °C. Li added GDC achieve more than 95 percent densification while Zn and Cu added GDC were only 90 percent densified (refer to Fig. 27).

**Fig. 28:**
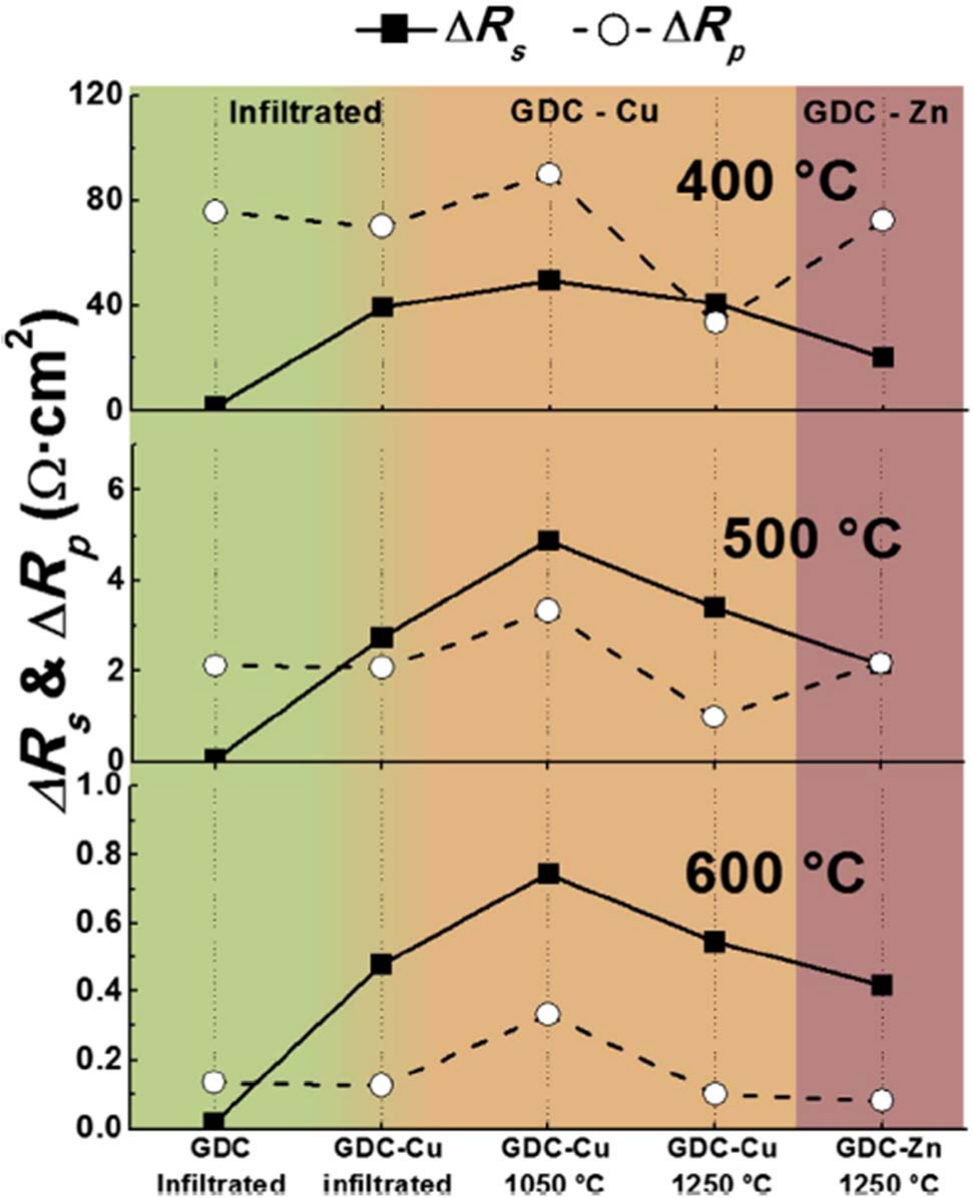
Comparison of Δ*R*_s_ and Δ*R*_p_ at 400 °C, 500 °C and 600 °C for all cells, depending on the preparation method of the GDC interlayer. (Reproduced with permission from ref. 31 Copyright 2017 Elsevier.)

Although sintering aids can help achieve the required relative, additional interfacial reactions occur which eventually affect the durability of cathodes and fuel cells. This group realized the adverse effects of using sintering aids hence devised an infiltration method to mitigate the additional reaction that may occur during fuel cell operation. The strategy was to screen-print a pre-sintered GDC and then infiltrate it with Ce and Gd nitrate solution. This method not only produced the densest layer of GDC but also showed low polarization resistance as seen in Fig. 28. It was reported that cell fabricated using the infiltrated exhibited at least 40% higher performance than the one fabricated with commercial GDC.

#### 4.3.2. Layered Bi_2_O_3_

Although infiltration offers a promising route for densification of interlayers but there are other techniques that lower the sintering temperature using sintering aids that do not promote other interfacial reactions. Lee *et al.*^30^ found an interesting material to be used as sintering aid. Instead of the conventional technique of doping or lattice penetration used, layered bismuth oxide (Bi_2_O_3_) was placed on the top of GDC to facilitate sinterability. Since it is not doped, the oxide layer on the top turns into liquid at roughly 800 °C. This helps GDC to form grains and densify without a decrease in ionic conductivity since the sintering aids sublimes at 1000 °C leaving pure, densified GDC.

**Fig. 29:**
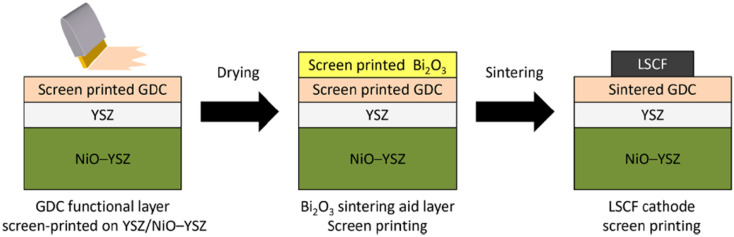
Fabrication route of deposition of sintering aid (Bi_2_O_3_) on GDC interlayer. (Reproduced with permission from ref. 30 Copyright 2022 Elsevier.)

**Fig. 30:**
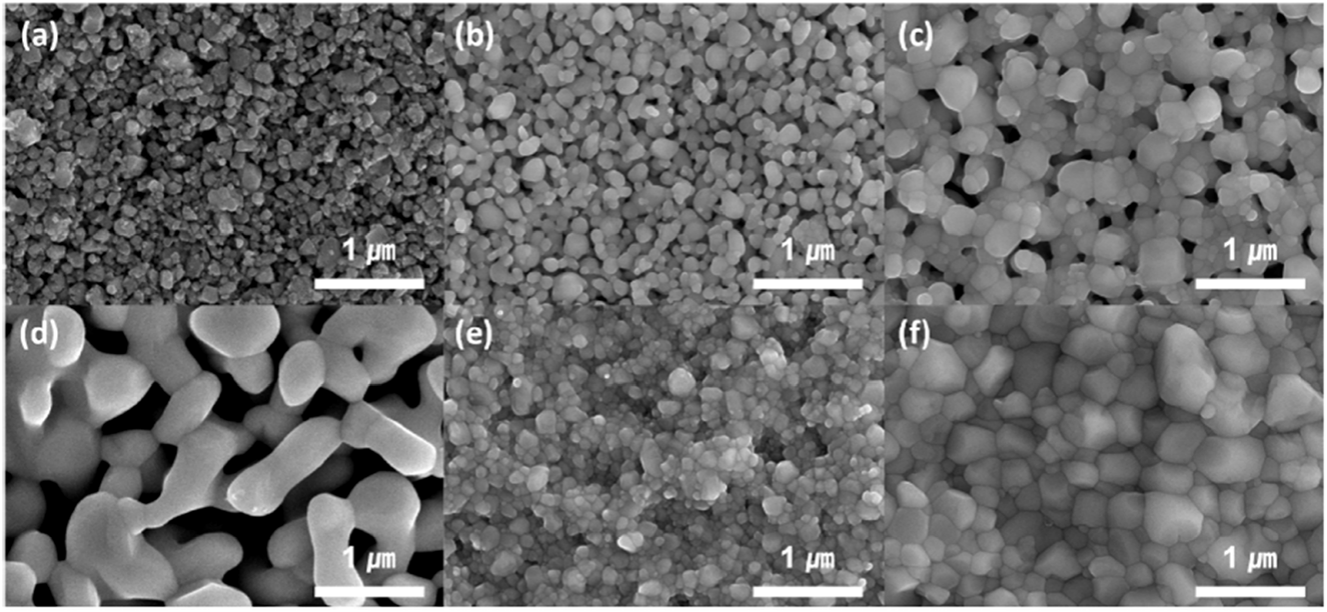
FE-SEM imaging of GDC after screen printing: (a) dried at 500 °C, (b) sintered at 1000 °C, and (c) sintered at 1200 °C; GDC with bismuth sintering aid: (d) dried at 500 °C, (e) sintered at 1000 °C, and (f) sintered at 1200 °C. (Reproduced with permission from ref. 30 Copyright 2022 Elsevier.)

**Fig. 31:**
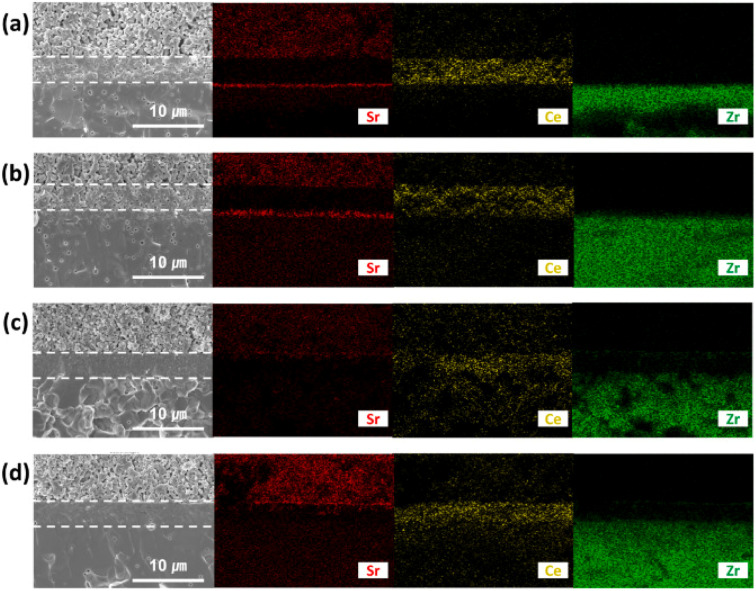
EDS mapping of cross-section side of GDC: (a) sintered at 1000 °C and (b) 1200 °C; GDC with Bi_2_O_3_ layer (c) sintered at 1000 °C and (d) 1200 °C. (Reproduced with permission from ref. 30 Copyright 2022 Elsevier.)

This group fabricated anode supported solid oxide cells using by first screen-printing GDC and then the oxide layer. It was then sintered and finally LSCF cathode was applied as illustrated in the Fig. 29. The FE-SEM images show a very dense GDC layer at 1200 °C (refer to Fig. 30). Moreover, this highly dense GDC was able to suppress strontium segregation and the secondary phases as depicted in the EDS attached in Fig. 31.

#### 4.3.3. Physical vapor deposition

**Fig. 32:**
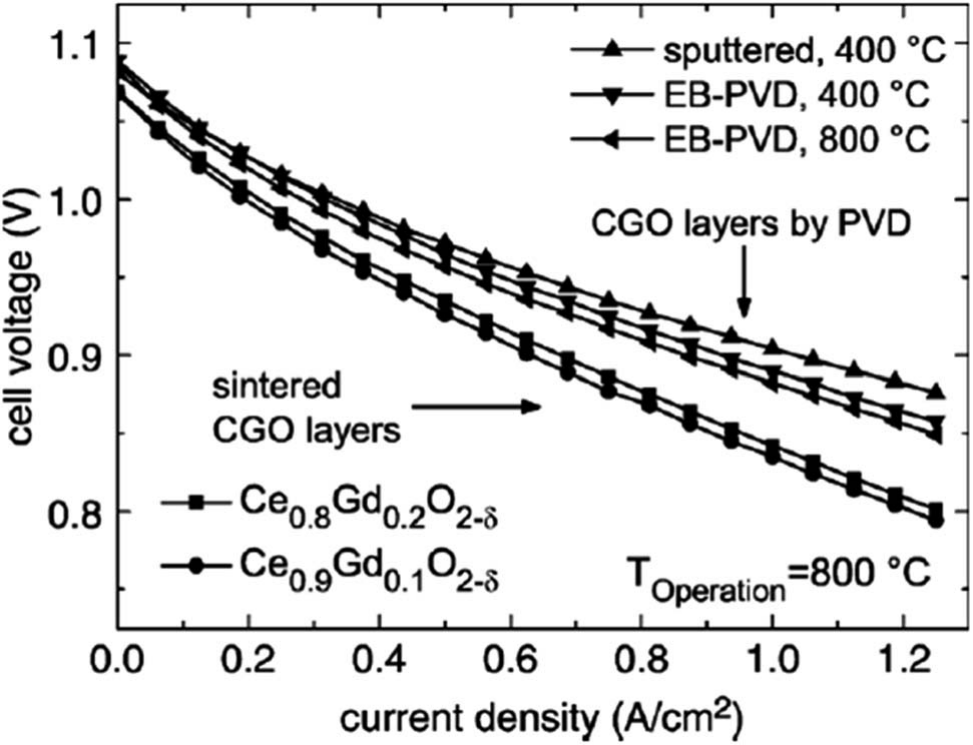
*I*–*V* curves of cell with sintered and physical-vapor-deposited GDC buffer layers run at 800 °C. (Reproduced with permission from ref. 84 Copyright 2007 Elsevier.)

Although the use of sintering aid is a common way to densify GDC, the exploitation of novel deposition techniques can also help densify the interlayer without promoting interfacial reactions with adjacent components of SOFC. Uhlenbruck *et al.*^84^ investigated the effectiveness of Physical Vapor Deposition (PVD) as compared to the conventional screen printing of GDC interlayer on the SOFC single cell anode -supported assembly. The cell with GDC deposited using PVD showed better electrochemical performance in comparison to the cell with screen-printed GDC as shown by the voltage–current density curves in Fig. 32.

#### 4.3.4. Ion assisted deposition

**Fig. 33:**
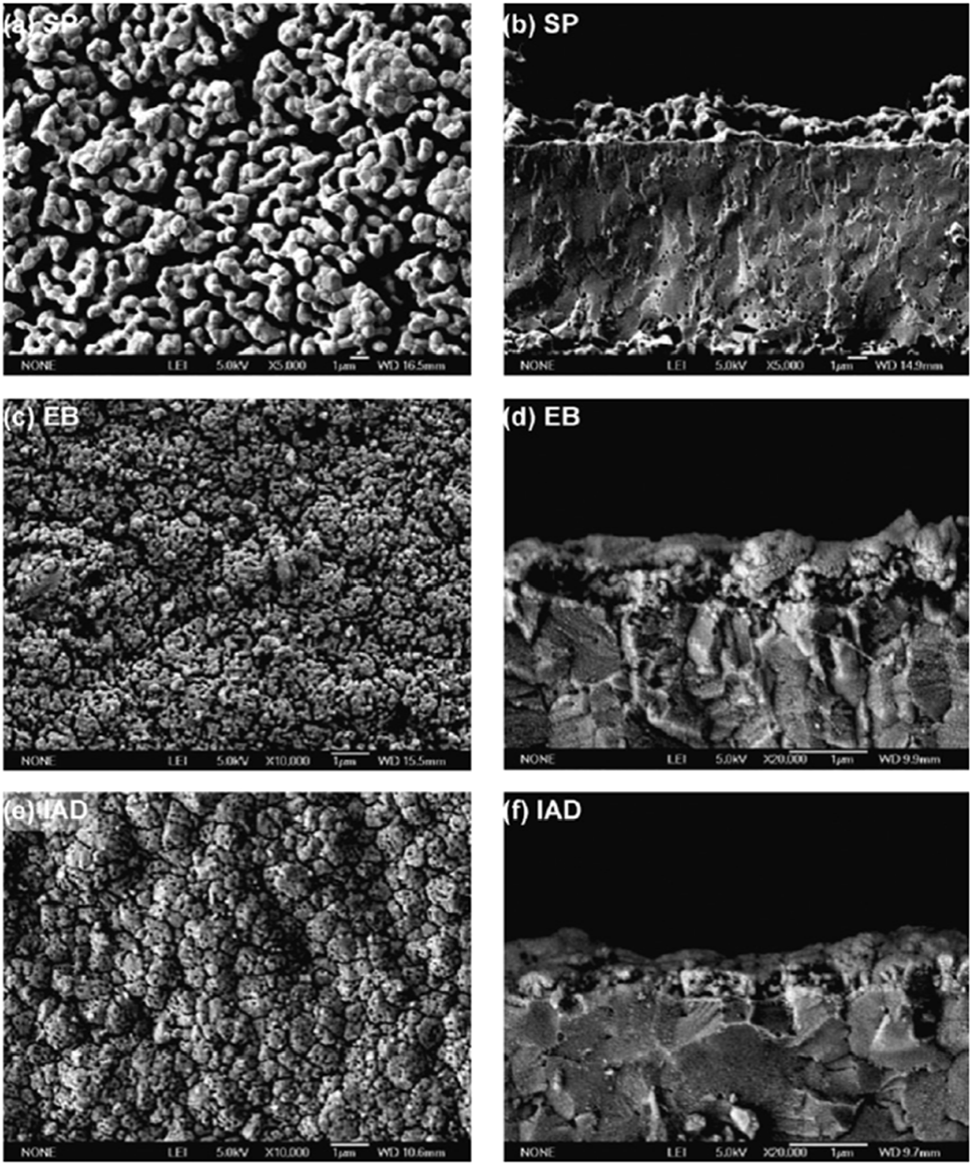
Microstructures of GDC interlayers: (a and b) screen printed GDC; (c and d) electron beam deposited GDC; (e and f) ion-assisted deposited GDC. Reproduced with permission from ref. 85 Copyright 2011 Elsevier.

Although physical vapor deposition shows reasonable results in comparison to the conventional screen printing for application of dense GDC, there are some other techniques explored by researchers like Gong *et al.*^85^ in a short communication, this group used screen printing, electron beam evaporation and ion assisted deposition to prepare GDC interlayer. There was a definite correlation between the deposition and the microstructure and relative density of the ceria-based interlayer. The screen-printing technique produced a porous microstructure. The use of EB and IAD greatly improved the microstructure characteristics of GDC interlayer as show by surface and cross-sectional images in Fig. 33.

The microstructure resulting from EB looks promising but did not result in good electrochemical performance. Firstly, the density was not enough to successfully suppress elemental migration from cathode bulk to cathode/GDC interface. Secondly, the adhesion between the electrolyte and GDC was poor.

**Fig. 34:**
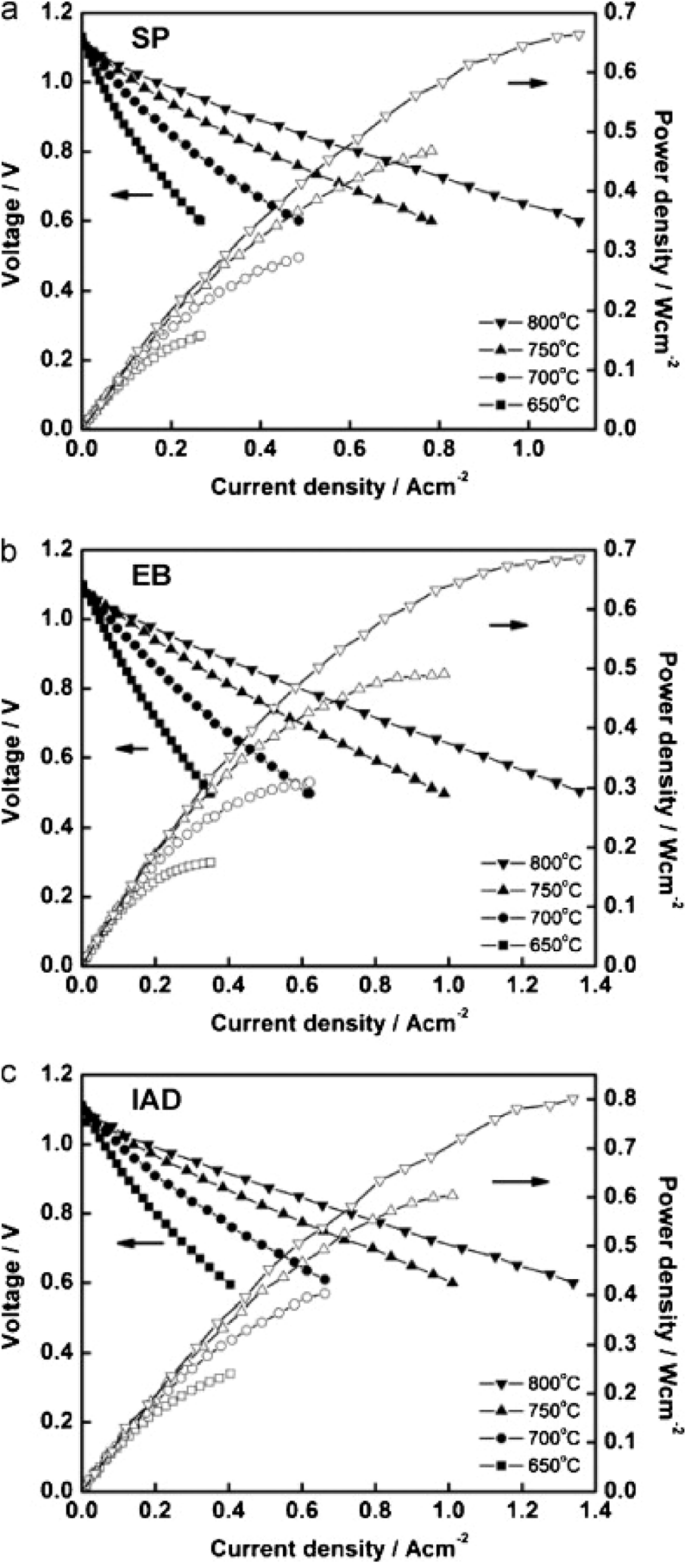
*I*–*V* curves of cells fabricated from GDC deposited by (a) screen printing (b) electron beam evaporation (c) ion assisted deposition. Reproduced with permission from ref. 85 Copyright 2011 Elsevier.

However, the IAD techniques solved these problems to a great extent. The contact improves between YSZ and GDC and from Fig. 34, we can see improved electrochemical performance as well.

## 5. Conclusion

This review aimed to summarize three major degradation problems of LSCF/LSC cathode in an anode-supported SOFC. Element segregation at the cathode surface like strontium, interaction of Cr-vapor from metallic interconnect with cathode surface and secondary phases and cation interdiffusion at the YSZ/GDC and GDC/LSCF interface were discussed. As the concluding remarks of this review paper, we present pointers of our review study.

• Strontium segregation is rampant issue that worsen in SOFC if operated for 1000 h at critical current density. The interaction of Sr with oxygen vacancies at the surface of the cathode needs in-depth study to be properly understood. An optimum level of oxygen vacancies leads to controlled Sr segregation which is beneficial for the ORR and long-term durability of SOFC.

• Cr-containing metallic interconnects like SUS430 release Cr-vapor as electrochemical driving force exist in the system due to cathode polarization. It can be effective mitigated by means of protective coatings like Mn-based thin spinel.

• The use of ceria-based interlayer requires trade-off research for the optimization of relative density and sintering temperature. Many researchers have tried lowering the temperature of the sintering process and applied several methods which proved effective. However, the long-term durability of these solution remains an area unexplored.

## 6. Prospects

In view of the degradation problems, efforts have been made to extend the lifetime of SOFC power systems. From the dawn of SOFC in 1937 to the first scientific assessment in 1962 by Weissbart and Ruka^124^ to the continuous research & development effort being put in, SOFC has huge potential to become an effective, robust power generation technology. It has many prospective applications in transportation, portable and auxiliary power units. The current market has set out for the development of kW-scale units to be used in residential and other large-scale applications. The key issue, as highlighted in the start, is extending lifetime up to 40 000 h with minimal degradation. Among the planar SOFCs,

• Siemens Westinghouse Power (SWH) first developed a 10 kW stack with 80 cells placed between bipolar plates.^125^

• Chubu Electric Power Company and Mitsubishi Heavy Industries obtained a power density of 0.22 W cm^−2^ with 5 kW planar SOFC.^125^

• In Australia, Ceramic Fuel Cells fabricated 400 cells and stacked them into a 5 kW system.^126^

More recently,

• Bloom energy has built a SOFC stack of 40 cells on natural gas feed; it claimed to have sold 80.9 MW of SOFC units cumulatively until 2018.^127^

• In 2016, MHI tested a hybrid system of SOFC and gas turbine; they reported fuel flexibility and 55 percent generation efficiency of their system.^128^

• In 2020, an Austria-based company Elcogen joined hands with Magnex CO (Japanese) to commercialize SOFC products.^129^

• Ceres Power (UK) also signed contracts with Robert Bosch (Germany) and Doosan (Korea) to developed kW-class stacks.^130^

• Magnex CO, on the other hand, is currently developing a 1 kW stack & 250 W portable system.^129^

To sum up these efforts, they are driven by two major factors: reducing cost and extending lifetime of SOFC stacks. The materials that can withstand high temperatures are either limited or costly and research is required to fabricate or modify existing material into durable systems. The current market goal is to alleviate degradation problems, extend lifetime (up to 40 000 h), make SOFC an affordable power generation technology; such a target will be achieved only with continuous research and innovation.

## Conflicts of interest

There are no conflicts to declare.

## Supplementary Material
